# Dual signaling pathways of TGF-β superfamily cytokines in hepatocytes: balancing liver homeostasis and disease progression

**DOI:** 10.3389/fphar.2025.1580500

**Published:** 2025-04-07

**Authors:** Roohi Chaudhary, Ralf Weiskirchen, Marcelo Ehrlich, Yoav I. Henis

**Affiliations:** ^1^ Shmunis School of Biomedicine and Cancer Research, George S. Wise Faculty of Life Sciences, Tel Aviv University, Tel Aviv, Israel; ^2^ Department of Neurobiology, George S. Wise Faculty of Life Sciences, Tel Aviv University, Tel Aviv, Israel; ^3^ Institute of Molecular Pathobiochemistry, Experimental Gene Therapy and Clinical Chemistry (IFMPEGKC), RWTH University Hospital Aachen, Aachen, Germany

**Keywords:** TGF-β superfamily, hepatocytes, SMAD signaling, non-canonical pathways, MASLD, MASH, hepatocellular carcinoma, liver fibrosis

## Abstract

The transforming growth factor-β (TGF-β) superfamily (TGF-β-SF) comprises over 30 cytokines, including TGF-β, activins/inhibins, bone morphogenetic proteins (BMPs), and growth differentiation factors (GDFs). These cytokines play critical roles in liver function and disease progression. Here, we discuss Smad-dependent (canonical) and non-Smad pathways activated by these cytokines in a hepatocellular context. We highlight the connection between the deregulation of these pathways or the balance between them and key hepatocellular processes (e.g., proliferation, apoptosis, and epithelial-mesenchymal transition (EMT)). We further discuss their contribution to various chronic liver conditions, such as metabolic dysfunction-associated steatotic liver disease (MASLD), metabolic dysfunction-associated steatohepatitis (MASH), and hepatocellular carcinoma (HCC). In MASLD and MASH, TGF-β signaling contributes to hepatocyte lipid accumulation, cell death and fibrosis progression through both Smad and non-Smad pathways. In HCC, TGF-β and other TGF-β-SF cytokines have a dual role, acting as tumor suppressors or promoters in early vs. advanced stages of tumor progression, respectively. Additionally, we review the involvement of non-Smad pathways in modulating hepatocyte responses to TGF-β-SF cytokines, particularly in the context of chronic liver diseases, as well as the interdependence with other key pathways (cholesterol metabolism, insulin resistance, oxidative stress and lipotoxicity) in MASLD/MASH pathogenesis. The perspectives and insights detailed in this review may assist in determining future research directions and therapeutic targets in liver conditions, including chronic liver diseases and cancer.

## 1 Introduction

The transforming growth factor-β (TGF-β) superfamily (TGF-β-SF; over 30 cytokines) includes TGF-βs, activins/inhibins, bone morphogenetic proteins (BMPs), growth and differentiation factors (GDFs), Müllerian inhibiting substance and nodals (Massagué, 2012; Morikawa et al., 2016; Luo, 2017). They are critical for multiple physiological and pathological processes, including several types of cancer, fibrosis, apoptosis, skeletal and vascular diseases, primary pulmonary hypertension, and angioproliferative disorders (Katz et al., 2013; Meng et al., 2016; Goumans et al., 2018; Allen et al., 2020; Marvin et al., 2020). They signal via heterotetrameric complexes of type II/type I dual-specificity (Ser/Thr and Tyr) kinase receptors (Ehrlich et al., 2012; Goebel et al., 2019), activating canonical Smad (Smad2/3 or Smad1/5/8) and non-Smad pathways, whose crosstalk is crucial for multiple responses (Parvani et al., 2011; Mu et al., 2012; Luo, 2017; Tzavlaki and Moustakas, 2020; Huang et al., 2022). TGF-β-SF signaling has been shown to play diverse roles in a variety of human diseases, including cancer.

Liver function and disease progression are important targets for TGF-β-SF cytokines, which regulate the balance between key hepatocellular processes, including proliferation, apoptosis, and epithelial-mesenchymal transition (EMT) (Xu et al., 2009; Schon and Weiskirchen, 2014; Fabregat et al., 2016; Zhang, 2017; Fabregat and Caballero-Díaz, 2018; Dewidar et al., 2019; Wang X. L. et al., 2024). Accordingly, several TGF-β-SF cytokines are expressed and/or secreted in specific hepatic cell populations, with their expression being most visible during liver disease (Wang X. L. et al., 2024). These include TGF-βs (Baer et al., 1998; Jeong et al., 2004; Dropmann et al., 2016; Zou et al., 2021; Deng et al., 2024), activins (Lau et al., 2000; Chabicovsky et al., 2003; Rodgarkia-Dara et al., 2006; Deli et al., 2008), BMPs (Herrera et al., 2014; Chung et al., 2018; Vacca et al., 2020) and GDF15 (Desmedt et al., 2019; Qi et al., 2021; Galuppo et al., 2022; Jurado-Aguilar et al., 2024; Li et al., 2024). Recent reviews have thoroughly described TGF-β (but not other TGF-β-SF cytokines) in the context of oncogenesis and fibrosis (Dooley and ten Dijke, 2012; Giannelli et al., 2016b; Fabregat and Caballero-Díaz, 2018; Dewidar et al., 2019). In this review, we focus on the balance between the signaling of multiple TGF-β-SF cytokines via Smad-dependent vs. non-Smad pathways and their role in chronic liver conditions, including metabolic dysfunction-associated steatotic liver disease (MASLD), metabolic dysfunction-associated steatohepatitis (MASH) and hepatocellular carcinoma (HCC) (Dooley and ten Dijke, 2012; Katz et al., 2016; Dewidar et al., 2019; Rao and Mishra, 2019; Marvin et al., 2020; Nair and Nath, 2020; Gonzalez-Sanchez et al., 2021). The review also discusses the impact of crosstalk between TGF-β-SF signaling and cellular processes such as cholesterol metabolism (Chaudhary et al., 2024; Wang S. et al., 2024), insulin resistance (Hong et al., 2016; Feng et al., 2024), oxidative stress (Yang et al., 2014; Yang et al., 2017; Chen et al., 2020) and lipotoxicity (Yamaguchi et al., 2022).

## 2 The role of the TGF-β superfamily in liver function and disease

### 2.1 TGF-βs

TGF-βs play a multifaceted role in liver physiology and pathology, with recent findings suggesting their contributions to liver fibrosis (Table 1), MASLD/MASH (Table 2), and HCC (Table 3). There are three human TGF-β isoforms (TGF-β1, 2, 3). Their expression in the liver is not high in homeostasis, but can be significantly increased in disease (Baer et al., 1998; Dropmann et al., 2016; Deng et al., 2024). Changes in expression levels may result from differential activation of different cell types, as well as differences in the relative abundance of such cells. In the latter context, immune and matrix cells of the liver (e.g., Kupffer cells and fibroblasts) intrinsically exhibit higher levels of expression of TGF-β1 than hepatocytes (Karlsson et al., 2021). TGF-β has a dual-faceted function in HCC, acting either as a tumor suppressor or promoter, depending on the disease stage (see section 4.2). Recent findings focus on the contribution of TGF-β signaling to liver fibrosis, raising possibilities to define novel therapeutic targets (Giannelli et al., 2016b; Dewidar et al., 2019; Bertolio et al., 2021; Liu X. et al., 2024; Crouchet et al., 2025). Thus, TβRII-SE, a newly identified soluble isoform of the TGF-β type II receptor that binds all three TGF-β isoforms, was shown to reduce liver injury and fibrosis in a carbon tetrachloride-induced liver fibrosis animal model, suggesting its potential as a therapeutic agent in fibrotic liver diseases (Bertolio et al., 2021). Additionally, a novel TGF-β type I receptor-mimicking peptide variant (Z-RIPΔ) was found to selectively target activated hepatic stellate cells (HSCs), inhibiting their proliferation and migration, downregulating fibrosis markers, and blocking TGF-β1-mediated signaling pathways (Liu X. et al., 2024). The relevance of TGF-β to other liver pathogenic conditions is exemplified in fatty liver conditions such as MASLD/MASH (Table 2), where latent TGF-β1 was shown to be produced and activated by macrophages through inflammatory cytokines (Ishiyama et al., 2024). This drives HSC activation and collagen (COL1A1) production, highlighting a specific mechanism for fibrosis progression in fatty liver disease (Ishiyama et al., 2024). Interestingly, recent studies on the effect of the circadian clock on liver fibrosis (Crouchet et al., 2025) showed that the circadian clock temporally gates TGF-β signaling, and this regulation is broken in fibrosis. As TGF-β signaling alleviated liver fibrosis in patient-derived models, this axis may offer potential therapeutic targets (Crouchet et al., 2025).

**TABLE 1:** TGF-β superfamily in liver fibrosis**.**

S.N.	Title	Type of article	References
1	Transforming growth factor-β-induced cell plasticity in liver fibrosis and hepatocarcinogenesis	Review	Fabregat and Caballero-Díaz (2018)
2	TGF-β in hepatic stellate cell activation and liver fibrogenesis-Updated 2019	Review	Dewidar et al. (2019)
3	Roles of transforming growth factor-β signaling in liver disease	Review	Wang, X. L. et al. (2024)
4	TGF-β in progression of liver disease	Review	Dooley and ten Dijke (2012)
5	Prevention of TGF-β-induced early liver fibrosis by a maleic acid derivative anti-oxidant through suppression of ROS, inflammation and hepatic stellate cells activation	Research paper	Yang et al. (2017)
6	The rationale for targeting TGF-β in chronic liver diseases	Review	Giannelli et al. (2016b)
7	A novel splice variant of human TGF-β type II receptor encodes a soluble protein and its Fc-tagged version prevents liver fibrosis *in vivo*	Research paper	Bertolio et al. (2021)
8	Targeting the liver clock improves fibrosis by restoring TGF-β signaling	Research paper	Crouchet et al. (2025)
9	Targeting delivery of a novel TGF-β type I receptor-mimicking peptide to activated hepatic stellate cells for liver fibrosis therapy via inhibiting the TGF-β1/Smad and p38 MAPK signaling pathways	Research paper	Liu, X. et al. (2024)
10	Assessing the combined impact of fatty liver-induced TGF-β1 and LPS-activated macrophages in fibrosis through a novel 3D serial section methodology	Research paper	Ishiyama et al. (2024)
11	ECM1 prevents activation of transforming growth factor β, hepatic stellate cells, and fibrogenesis in mice	Research paper	Fan et al. (2019)
12	ECM1 attenuates hepatic fibrosis by interfering with mediators of latent TGF-β1 activation	Research paper	Link et al. (2024)
13	TGF-β1 and TGF-β2 abundance in liver diseases of mice and men	Research paper	Dropmann et al. (2016)
14	Orphan nuclear receptor ERRγ regulates hepatic TGF-β2 expression and fibrogenic response in CCl_4_-induced acute liver injury	Research paper	Jung et al. (2021)
15	TGF-β and HIPPO signaling pathways interplay in distinct hepatic contexts	Review	Color-Aparicio et al. (2024)
16	The activin axis in liver biology and disease	Review	Rodgarkia-Dara et al. (2006)
17	Gastrointestinal pharmacology activins in liver health and disease	Review	Hamang et al. (2023)
18	Antagonizing activin A/p15(INK4b) signaling as therapeutic strategy for liver disease	Research paper	Mekala et al. (2024)
19	Potential roles of BMP9 in liver fibrosis	Review	Bi and Ge (2014)
20	BMP signalling at the crossroad of liver fibrosis and regeneration	Review	Herrera et al. (2018)
21	New insights into BMP9 signaling in organ fibrosis	Review	Tang et al. (2020)
22	Unveiling the impact of BMP9 in liver diseases: Insights into pathogenesis and therapeutic potential	Review	Chen et al. (2024)
23	BMP-9 interferes with liver regeneration and promotes liver fibrosis	Research paper	Breitkopf-Heinlein et al. (2017)
24	Bone morphogenetic protein-7 represses hepatic stellate cell activation and liver fibrosis via regulation of TGF-β/Smad signaling pathway	Research paper	Zou et al. (2019)
25	GDF15 deficiency exacerbates chronic alcohol- and carbon tetrachloride-induced liver injury	Research paper	Chung et al. (2017)
26	Growth differentiation factor 15: Emerging role in liver diseases	Review	Li et al. (2024)
27	GDF15 ameliorates liver fibrosis by metabolic reprogramming of macrophages to acquire anti-inflammatory properties	Research paper	Li et al. (2023)
28	Glial cell line-derived neurotrophic factor (GDNF) mediates hepatic stellate cell activation via ALK5/Smad signalling	Research paper	Tao et al. (2019)
29	Expression and function of BMP and activin membrane-bound inhibitor (BAMBI) in chronic liver diseases and hepatocellular carcinoma	Review	Weber et al. (2023)
30	MicroRNA-942 mediates hepatic stellate cell activation by regulating BAMBI expression in human liver fibrosis	Research paper	Tao et al. (2018)
31	Transforming growth factor-β signaling in hepatocytes promotes hepatic fibrosis and carcinogenesis in mice with hepatocyte-specific deletion of TAK1	Research paper	Yang et al. (2013)
32	REDD1 attenuates hepatic stellate cell activation and liver fibrosis via inhibiting of TGF-β/Smad signaling pathway	Research paper	Cho et al. (2021)
33	Transforming growth factor-β (TGF-β) directly activates the JAK1-STAT3 axis to induce hepatic fibrosis in coordination with the SMAD pathway	Research paper	Tang et al. (2017)
34	Orphan nuclear receptor NR4A1 regulates transforming growth factor-β signaling and fibrosis	Research paper	Palumbo-Zerr et al. (2015)
35	The miR-3074/BMP7 axis regulates TGF-β-caused activation of hepatic stellate cells *in vitro* and CCl_4_-caused murine liver fibrosis *in vivo*	Research paper	Liu, B. et al. (2024)
36	Epithelial transforming growth factor-β signaling does not contribute to liver fibrosis but protects mice from cholangiocarcinoma	Research paper	Mu et al. (2016)
37	Id1 is a critical mediator in TGF-β-induced transdifferentiation of rat hepatic stellate cells	Research paper	Wiercinska et al. (2006)
38	A gut microbial metabolite of linoleic acid ameliorates liver fibrosis by inhibiting TGF-β signaling in hepatic stellate cells	Research paper	Kasahara et al. (2023)
39	Genome-wide CRISPR screening to identify drivers of TGF-β-induced liver fibrosis in human hepatic stellate cells	Research paper	Yu et al. (2022)
40	Hepatocyte FoxO1 deficiency protects from liver fibrosis via reducing inflammation and TGF-β1-mediated HSC activation	Research paper	Pan et al. (2024)
41	Can a fibrotic liver afford epithelial-mesenchymal transition?	Review	Munker et al. (2017)
42	Tyrosine kinase receptor B attenuates liver fibrosis by inhibiting TGF-β/SMAD signaling	Research paper	Song et al. (2023)
43	Gli2-regulated activation of hepatic stellate cells and liver fibrosis by TGF-β signaling	Research paper	Yan et al. (2021)
44	Maresin-1 prevents liver fibrosis by targeting Nrf2 and NF-κB, reducing oxidative stress and inflammation	Research paper	Rodríguez et al. (2021)
45	Oxy210, a novel inhibitor of hedgehog and TGF-β signalling, ameliorates hepatic fibrosis and hypercholesterolemia in mice	Research paper	Hui et al. (2021)
46	Inhibition of TGFβ type I receptor activity facilitates liver regeneration upon acute CCl_4_ intoxication in mice	Research paper	Karkampouna et al. (2016)

**TABLE 2:** TGF-β superfamily in MASLD/MASH**.**

S.N.	Title	Type of article	References
1	Inevitable role of TGF-β1 in progression of nonalcoholic fatty liver disease	Review	Nair and Nath (2020)
2	TGF-β signaling in hepatocytes participates in steatohepatitis through regulation of cell death and lipid metabolism	Research paper	Yang et al. (2014)
3	Smad3 phospho-isoform signaling in nonalcoholic steatohepatitis	Review	Yamaguchi et al. (2022)
4	Roles of transforming growth factor-β signaling in liver disease	Review	Wang, X. L. et al. (2024)
5	TGF-β in progression of liver disease	Review	Dooley and ten Dijke (2012)
6	The rationale for targeting TGF-β in chronic liver diseases	Review	Giannelli et al. (2016b)
7	Assessing the combined impact of fatty liver-induced TGF-β1 and LPS-activated macrophages in fibrosis through a novel 3D serial section methodology	Research paper	Ishiyama et al. (2024)
8	ECM1 modified HF-MSCs targeting HSC attenuate liver cirrhosis by inhibiting the TGF-β/Smad signaling pathway	Research paper	Liu et al. (2023b)
9	TGF-β1 and TGF-β2 abundance in liver diseases of mice and men	Research paper	Dropmann et al. (2016)
10	TGF-β and HIPPO signaling pathways interplay in distinct hepatic contexts	Review	Color-Aparicio et al. (2024)
11	The activin axis in liver biology and disease	Review	Rodgarkia-Dara et al. (2006)
12	A complex role of activin A in non-alcoholic fatty liver disease	Research paper	Yndestad et al. (2009)
13	Gastrointestinal pharmacology activins in liver health and disease	Review	Hamang et al. (2023)
14	Roles of activin A and Gpnmb in metabolic dysfunction-associated steatotic liver disease (MASLD)	Research paper	Liu, H. et al. (2023b)
15	Unveiling the impact of BMP9 in liver diseases: Insights into pathogenesis and therapeutic potential	Review	Chen et al. (2024)
16	Circulating bone morphogenetic protein 9 (BMP9) as a new biomarker for noninvasive stratification of nonalcoholic fatty liver disease and metabolic syndrome	Research paper	Yang et al. (2024)
17	The role of bone morphogenetic protein 9 in nonalcoholic fatty liver disease in mice	Research paper	Sun et al. (2021)
18	Adenovirus-mediated overexpression of bone morphogenetic protein-9 promotes methionine choline deficiency-induced non-alcoholic steatohepatitis in non-obese mice	Research paper	Li et al. (2019)
19	Bone morphogenetic protein 4 alleviates nonalcoholic steatohepatitis by inhibiting hepatic ferroptosis	Research paper	Wang et al. (2022)
20	Growth differentiation factor 15 (GDF15) is associated with non-alcoholic fatty liver disease (NAFLD) in youth with overweight or obesity	Research paper	Galuppo et al. (2022)
21	Growth differentiation factor 15: Emerging role in liver diseases	Review	Li et al. (2024)
22	Hepatocyte-specific GDF15 overexpression improves high-fat diet-induced obesity and hepatic steatosis in mice via hepatic FGF21 induction	Research paper	Takeuchi et al. (2024)
23	Elevated serum growth differentiation factor 15 and decorin predict the fibrotic progression of metabolic dysfunction-associated steatotic liver disease	Research paper	Chang et al. (2024)
24	Expression and function of BMP and activin membrane-bound inhibitor (BAMBI) in chronic liver diseases and hepatocellular carcinoma	Review	Weber et al. (2023)
25	Multispecies transcriptomics identifies SIKE as a MAPK repressor that prevents NASH progression	Research paper	Bai et al. (2024)
26	TGF-β1 signaling can worsen NAFLD with liver fibrosis backdrop	Review	Ahmed et al. (2022)
27	Pathogenesis of non-alcoholic fatty liver disease mediated by YAP.	Research paper	Chen, P. et al. (2018)
28	TNFAIP3 interacting protein 3 overexpression suppresses nonalcoholic steatohepatitis by blocking TAK1 activation	Research paper	Liu et al. (2020)
29	Promotion of liver regeneration and anti-fibrotic effects of the TGF-β receptor kinase inhibitor galunisertib in CCl_4_-treated mice	Research paper	Masuda et al. (2020)

**TABLE 3:** TGF-β superfamily in liver cancer/HCC.

S.N	Title	Type of article	References
1	TGF-β signaling in liver and gastrointestinal cancers	Review	Katz et al. (2013)
2	TGF-β signaling in liver metastasis	Review	Marvin et al. (2020)
3	Transforming growth factor-β-induced cell plasticity in liver fibrosis and hepatocarcinogenesis	Review	Fabregat and Caballero-Díaz (2018)
4	Roles of transforming growth factor-β signaling in liver disease	Review	Wang, X. L. et al. (2024)
5	TGF-β in progression of liver disease	Review	Dooley and ten Dijke (2012)
6	Targeting transforming growth factor β signaling in liver cancer	Review	Rao and Mishra (2019)
7	The TGF-β pathway: A pharmacological target in hepatocellular carcinoma?	Review	Gonzalez-Sanchez et al. (2021)
8	The rationale for targeting TGF-β in chronic liver diseases	Review	Giannelli et al. (2016b)
9	TGF-β1 and TGF-β2 abundance in liver diseases of mice and men	Research paper	Dropmann et al. (2016)
10	TGF-β and HIPPO signaling pathways interplay in distinct hepatic contexts	Review	Color-Aparicio et al. (2024)
11	The activin axis in liver biology and disease	Review	Rodgarkia-Dara et al. (2006)
12	Activins and activin antagonists in hepatocellular carcinoma	Review	Deli et al. (2008)
13	Activins and follistatins: Emerging roles in liver physiology and cancer	Review	Kreidl et al. (2009)
14	Gastrointestinal pharmacology activins in liver health and disease	Review	Hamang et al. (2023)
15	Inhibin/activin expression in human and rodent liver: subunits a and bB as new players in human hepatocellular carcinoma?	Research paper	Frost et al. (2011)
16	Potential roles of bone morphogenetic protein (BMP)-9 in human liver diseases	Review	Herrera et al. (2014)
17	Unveiling the impact of BMP9 in liver diseases: Insights into pathogenesis and therapeutic potential	Review	Chen et al. (2024)
18	Growth differentiation factor 15: Emerging role in liver diseases	Review	Li et al. (2024)
19	Expression and function of BMP and activin membrane-bound inhibitor (BAMBI) in chronic liver diseases and hepatocellular carcinoma	Review	Weber et al. (2023)
20	SMAD4 exerts a tumor-promoting role in hepatocellular carcinoma	Research paper	Hernanda et al. (2015)
21	The role of TGF-β/SMAD signaling in hepatocellular carcinoma: from mechanism to therapy and prognosis	Review	Xin et al. (2024)
22	Smad7 regulates compensatory hepatocyte proliferation in damaged mouse liver and positively relates to better clinical outcome in human hepatocellular carcinoma	Research paper	Feng, X. et al. (2015)
23	Hepatic stem cells and transforming growth factor β in hepatocellular carcinoma	Review	Majumdar et al. (2012)
24	Transforming growth factor-β signaling in hepatocytes promotes hepatic fibrosis and carcinogenesis in mice with hepatocyte-specific deletion of TAK1	Research paper	Yang et al. (2013)
25	A transforming growth factor-β and H19 signaling axis in tumor-initiating hepatocytes that regulates hepatic carcinogenesis	Research paper	Zhang et al. (2019)
26	TGFβR1 inhibition drives hepatocellular carcinoma proliferation through induction of toll-like-receptor signaling	Research paper	Mohamed et al. (2024)
27	Epithelial transforming growth factor-β signaling does not contribute to liver fibrosis but protects mice from cholangiocarcinoma	Research paper	Mu et al. (2016)
28	Involvement of programmed cell death 4 in transforming growth factor-β1-induced apoptosis in human hepatocellular carcinoma	Research paper	Zhang et al. (2006)
29	Ethanol sensitizes hepatocytes for TGF-β-triggered apoptosis	Research paper	Gaitantzi et al. (2018)
30	Autophagy is activated by TGF-β and potentiates TGF-β-mediated growth inhibition in human hepatocellular carcinoma cells	Research paper	Kiyono et al. (2009)
31	Transforming growth factor-β induces senescence in hepatocellular carcinoma cells and inhibits tumor growth	Research paper	Senturk et al. (2010)
32	TGF-β/SMAD canonical pathway induces the expression of transcriptional cofactor TAZ in liver cancer cells	Research paper	Ríos-López et al. (2023)
33	TGF-β signaling in onset and progression of hepatocellular carcinoma	Review	Meindl-Beinker et al. (2012)
34	TGF-β and hepatocellular carcinoma: When a friend becomes an enemy	Review	Arrese et al. (2018)
35	Analysis of genomes and transcriptomes of hepatocellular carcinomas identifies mutations and gene expression changes in the transforming growth factor-β pathway	Research paper	Chen, J. et al. (2018)
36	Transforming growth factor-β promotes liver tumorigenesis in mice via upregulation of Snail	Research paper	Moon et al. (2017)
37	MFSD2A overexpression inhibits hepatocellular carcinoma through TGF-β/Smad Signaling	Research paper	Xiao et al. (2025)
38	The TGF-β1 target WISP1 is highly expressed in liver cirrhosis and cirrhotic HCC microenvironment and involved in pro- and anti-tumorigenic effects	Research paper	Dropmann et al. (2024)
39	Differential TGFβ pathway targeting by miR-122 in humans and mice affects liver cancer metastasis	Research paper	Yin et al. (2016)
40	Transforming growth factor-β drives the transendothelial migration of hepatocellular carcinoma cells	Research paper	Koudelkova et al. (2017)
41	The level of caveolin-1 expression determines response to TGF-β as a tumour suppressor in hepatocellular carcinoma cells	Research paper	Moreno-Càceres et al. (2017)
42	Cholesterol pathway inhibition induces TGF-β signaling to promote basal differentiation in pancreatic cancer	Research paper	Gabitova-Cornell et al. (2020)
43	Biomarkers and overall survival in patients with advanced hepatocellular carcinoma treated with TGF-βRI inhibitor galunisertib	Research paper	Giannelli et al. (2020)
44	Identification of EMT signaling cross-talk and gene regulatory networks by single-cell RNA sequencing	Research paper	Deshmukh et al. (2021)
45	TGF-β downstream of Smad3 and MAPK signaling antagonistically regulate the viability and partial epithelial-mesenchymal transition of liver progenitor cells	Research paper	Sun et al. (2024)
46	Novel transforming growth factor β receptor I kinase inhibitor galunisertib (LY2157299) in advanced hepatocellular carcinoma	Research paper	Faivre et al. (2019)
47	New and old key players in liver cancer	Review	Cuesta et al. (2023)
48	Galunisertib modifies the liver fibrotic composition in the Abcb4Ko mouse model	Research paper	Hammad et al. (2018)
49	Targeting TGF-beta I with the transforming growth factor receptor type I kinase inhibitor, LY2157299, modulates stemness-related biomarkers in hepatocellular carcinoma	Research paper	Rani et al. (2015)
50	A phase 2 study of galunisertib (TGF-β1 receptor type I inhibitor) and sorafenib in patients with advanced hepatocellular carcinoma	Research paper	Kelley et al. (2019)
51	Phase 1b study of galunisertib and ramucirumab in patients with advanced hepatocellular carcinoma	Research paper	Harding et al. (2021)

The regulatory roles of TGF-β in liver fibrosis are further exemplified through the phenotypes that arise from changes in the expression of proteins that control the availability of active TGF-β. For example, matrisomal proteins such as extracellular matrix protein 1 (ECM1) are crucial for depositing, stabilizing, and activating latent TGF-β (LTGF-β), a TGF-β precursor. ECM1 is critical for maintaining LTGF-β latency in the healthy liver, and its loss triggers harmful TGF-β signaling, damaging liver structure and function (Li et al., 2022). ECM1 also prevents TGF-β and HSCs activation and fibrogenesis in mice (Fan et al., 2019). A recent study showed that ECM1 knockout (KO) leads to LTGF-β1 activation, causing hepatic fibrosis and rapid mortality (Link et al., 2024). In ECM1-KO mouse liver tissue, LTGF-β1 activators such as thrombospondins (TSPs), ADAMTS proteases, and matrix metalloproteinases (MMPs), along with profibrotic gene expression, were upregulated (Link et al., 2024). Conversely, ECM1 overexpression in HSCs inhibited LTGF-β1 activation by these proteins. In patients with chronic liver disease (CLD), ECM1 expression negatively correlated with TSP-1, ADAMTS1 and MMP-2/9 levels, as well as with LTGF-β1 activation. These findings were supported by a computational model that outlines key interactions and cellular phenotypes in liver fibrogenesis (Link et al., 2024). Apart from HSCs, other cell types, such as myeloid cells, can be influenced by activated TGF-β to promote fibrosis. Studies in mice with a myeloid-specific deletion of the TGF-β type II receptor revealed that activated TGF-β and its regulation by hepatocytes are essential for activation of liver F4/80^+^/CD11b^hi^/CD14^hi^ macrophages through the C-C motif chemokine receptor (CCR2) (Wolf et al., 2023). This suggests that transient LTGF-β activation is essential in early liver regeneration and injury (Wolf et al., 2023).

The notion that ECM1 upregulation protects against liver fibrosis is further supported by the demonstration that genetically-induced overexpression of ECM1 in hair follicle-derived mesenchymal stem cells (HF-MSCs) improved their therapeutic potential for targeting cirrhosis. ECM1-overexpressing HF-MSCs significantly enhanced liver function, reduced liver injury, and inhibited HSC activation and TGF-β signaling to the Smad pathway (Liu et al., 2022). Another finding highlighted the role of plasma kallikrein-dependent LTGF-β activation in early fibrosis progression, revealing unique degradation products that could serve as novel surrogate markers to monitor TGF-β activity and CLD progression (Yokoyama et al., 2019). Recently, a distinct population of “liver-type” innate lymphoid cells (LT-ILC1s) has been characterized by cytokine production, lack of cytotoxic activity, and their expansion observed in cirrhotic liver tissues (Krämer et al., 2023). These LT-ILC1s are induced by TGF-β1 from blood-derived ILC precursors or liver sinusoidal endothelial cells *in vitro*, underscoring TGF-β′s role in shaping immune cell populations in liver fibrosis (Krämer et al., 2023).

Similar to TGF-β1, TGF-β2 plays a role in hepatic fibrogenesis (Dropmann et al., 2016). Recent findings have shown that TGF-β2 expression and secretion are mediated in a CCl_4_-induced liver injury model and depend on estrogen-related receptor γ (ERR_γ_) (Jung et al., 2021). IL-6 acts as an upstream signal to drive ERR_γ_ and TGF-β2 expression, with ERR_γ_ binding directly to the TGF-β2 promoter to regulate transcription. Inhibition of ERR_γ_ with the inverse agonist GSK5182 (a drug that binds to the agonist site but induces an inhibitory response) reduces TGF-β2 production, demonstrating that ERR_γ_ is a key regulator of TGF-β2-mediated fibrogenic responses in acute liver injury (Jung et al., 2021).

These findings underscore the pivotal role of TGF-β in liver function and disease progression, especially in fibrosis (Dewidar et al., 2019; Color-Aparicio et al., 2024; Wang X. L. et al., 2024) (Table 1). Thus, targeting TGF-β signaling through innovative strategies such as receptor mimetics, soluble receptors, or circadian modulation offers promising opportunities for therapeutic intervention.

### 2.2 Activins

Activins represent another branch of the TGF-β-SF. Activin A (ActA) was reported to play complex and context-dependent roles in liver physiology and pathology, with implications for liver regeneration, metabolic dysfunction, fibrosis, and diagnostics (Rodgarkia-Dara et al., 2006; Deli et al., 2008; Kreidl et al., 2009; Yndestad et al., 2009; Hamang et al., 2023). Studies using the newly developed ActA antagonist, NUCC-555, have shown dual benefits in promoting liver regeneration and halting fibrosis progression. In mouse and rat models of CLD, ActA induced overexpression of cell cycle and senescence-related genes, which were disrupted by NUCC-555 (Mekala et al., 2024). These findings suggest that blocking ActA signaling could offer therapeutic benefits in managing at least some chronic liver diseases. It is important to note that ActA has pleiotropic effects in the liver. Thus, under a different setting (*LDLR* ^*−/−*^ *in-vivo* mice model), ActA displayed protective effects against liver metabolic dysfunction (Liu, H. et al., 2023a). Here, opposite to the positive effects of ActA inhibition in chronic liver disease, ActA reduced inflammation, hematopoietic stem cell expansion, liver steatosis, and plasma cholesterol levels while diminishing atherosclerotic lesions (Liu, H. et al., 2023a). In another liver disease, MASLD, overexpression of hepatic ActA was found to counteract MASLD by reducing liver steatosis, systemic fat accumulation, and inflammation, while improving insulin sensitivity (Liu, H. et al., 2023b).

Taken together, these studies highlight the dual nature of ActA in liver biology, ranging from protective roles in metabolic liver diseases to pathological contribution in fibrosis, MASLD/MASH, and HCC pathogenesis (Tables 1–3). This underscores the need for further investigation to unravel ActA context-specific actions in order to harness its therapeutic and diagnostic potential in liver disorders.

### 2.3 Inhibins

Inhibins and their subunits play multifaceted roles in liver health, impacting metabolic regulation, insulin resistance, cancer progression, and fat distribution (Frost et al., 2011; Sugiyama et al., 2018; Deaton et al., 2022). Their diverse functions position them as promising targets for therapeutic intervention in various liver-related conditions.

Recent studies have highlighted Inhibin βE (INHBE) as a novel putative hepatokine linked to insulin resistance and obesity (Sugiyama et al., 2018). *INHBE* gene expression in the liver correlates positively with insulin resistance and body mass index in humans, and its levels are elevated in *db*/*db* mice, a rodent model of type 2 diabetes (Sugiyama et al., 2018). Knockdown of *INHBE* in these mice suppressed weight gain, reduced respiratory quotient and increased fat utilization (Sugiyama et al., 2018), suggesting that INHBE may be an emerging player in metabolic regulation and a potential target for the treatment of insulin resistance. Moreover, rare loss-of-function variants in the liver *INHBE* gene have been associated with reduced abdominal fat distribution, characterized by a lower waist-to-hip ratio (Deaton et al., 2022). These variants result in a substantial reduction in secreted INHBE levels, pointing it as a potential target for combatting abdominal obesity and associated metabolic disorders (Deaton et al., 2022).

In the context of liver cancer, inhibin subunits exhibit differential expression patterns. Inhibin α subunit, undetectable in normal liver tissue, is significantly upregulated in tumor-adjacent liver tissue and dramatically increased in HCC (Frost et al., 2011). Similarly, the expression of inhibin βB was elevated in certain HCC samples (Frost et al., 2011). This upregulation appears to shield HCC cells from the anti-proliferative effects of ActA, highlighting it as a potential mechanism promoting tumor progression and a possible target for therapeutic intervention (Frost et al., 2011).

Together, these findings underscore diverse and critical roles for inhibins in liver function and disease. Their involvement in metabolic regulation, insulin resistance, cancer progression, and fat distribution offers valuable insights into addressing liver-related metabolic and oncological conditions (Tables 1–3).

### 2.4 BMPs

BMPs form the largest subfamily of TGF-β-SF cytokines. Therefore, it is not surprising that they are also emerging as regulators of liver health and disease, with diverse roles in metabolic processes, fibrosis, and inflammation (Herrera et al., 2012; Bi and Ge, 2014; Herrera et al., 2014; Herrera et al., 2018; Tang et al., 2020) (Tables 1–3). Recent findings highlight the therapeutic and diagnostic potential of BMPs in liver disorders (Herrera et al., 2018; Chen et al., 2024). Thus, BMP9 was shown to serve as a potential biomarker and therapeutic target in MASLD and metabolic syndromes (Xu et al., 2017; Li et al., 2018; Chen et al., 2024; Yang et al., 2024). BMP9 levels were significantly reduced in MASLD mouse models while supplementing these animals with BMP9 improved their condition by downregulation of genes related to glucose or lipid metabolism, leading to reduced liver inflammation (Yang et al., 2024). In mice fed a high-fat diet (HFD), BMP9 alleviated obesity, improved glucose metabolism, and reduced hepatic steatosis (Yang et al., 2024). The mechanism appears to involve the effects of BMP9 on gene expression by reshaping chromatin accessibility, positioning it as a promising biomarker and therapeutic target for metabolic liver diseases. In line with these findings, ablation of BMP9 was reported to enhance liver steatosis, associated with the downregulation of peroxisome proliferator-activated receptor expression (Yang et al., 2020; Sun et al., 2021). On the other hand, BMP-9 can exacerbate methionine-choline-deficient diet-induced MASH in mice where its overexpression worsened liver inflammation, elevated serum alanine aminotransferase and aspartate transaminase, increased inflammatory gene expression, and enhanced M1 macrophage recruitment (Li et al., 2019). While BMP-9 overexpression did not affect pro-fibrogenic genes like *COL1A1* or *MMP9*, it upregulated TGF-β and plasminogen activator inhibitor 1 and downregulated *MMP2* expression, driving inflammation in MASH progression (Li et al., 2019).

BMP9 is also a negative regulator of oval cell expansion in cholestatic injury (Addante et al., 2018). Its deletion enhanced liver regeneration during 3,5 diethoxycarbonyl-1,4 dihydrocollidine (DDC)-induced cholestatic injury by inhibiting hepatic progenitor/oval cell expansion via activin-like kinase 2 (ALK2)-mediated Smad1/5/8 activation, reducing cell growth and promoting apoptosis. In addition, BMP9 deletion enhanced PI3K/AKT, ERK-MAPK and c-Met signaling, leading to increased ductular reaction, improved regenerative responses, reduced fibrosis, and decreased liver damage upon DDC feeding (Addante et al., 2018). BMP-9 is primarily produced by quiescent and activated HSCs and maintains hepatocyte function under normal conditions by inhibiting cell proliferation and EMT, while preserving metabolic enzyme expression. However, BMP-9 levels increase with HSC activation during liver injury, exacerbating damage in acute injury (e.g., partial hepatectomy) and promoting fibrosis in chronic liver injury (Breitkopf-Heinlein et al., 2017). In contrast, chronic liver injury in BMP-9-deficient mice or mice with adenoviral overexpression of the selective BMP-9 antagonist activin-like kinase 1-Fc demonstrated diminished collagen deposition and reduced fibrosis (Breitkopf-Heinlein et al., 2017). Thus, constitutive low expression of BMP-9 stabilized hepatocyte function in the healthy liver. Moreover, HSC activation was accompanied by increased endogenous BMP-9 levels *in vitro* and *in vivo*, and high levels of BMP-9 caused enhanced damage in acute or chronic injury (Breitkopf-Heinlein et al., 2017).

The expression level of another BMP cytokine, BMP4, was elevated in MASH models and was associated with reduced markers of ferroptosis and oxidative stress (Wang et al., 2022). These findings suggest that BMP4 may have a protective role in mitigating oxidative damage in MASH, highlighting its potential as a therapeutic agent. Moreover, during liver fibrosis progression, the levels of another BMP (BMP7) showed an initial increase followed by a decline in later stages. Exogenous BMP7 inhibited hepatic stellate cell activation, migration, and proliferation, with anti-fibrotic effects linked to the activation of the Smad1/5/8 pathway and suppression of Smad3 and p38 phosphorylation (Zou et al., 2019).

Hepatic *BMP6* gene expression is also transcriptionally regulated by the pro-inflammatory cytokine IL-6, which induces hepatic ERR_γ_ that binds to the BMP6 promoter and enhances its transcription (Radhakrishnan et al., 2020). ERR_γ_ knockdown in different cell lines or hepatocyte-specific KO *in vivo*, suppressed IL-6-driven BMP6 expression, while ERR_γ_ overexpression increased it (Radhakrishnan et al., 2020).

Mutations in ALK1 (*ACVRL1*), the receptor for BMP9/BMP10, are linked to severe liver vascular malformations in hereditary hemorrhagic telangiectasia (HHT). A novel HHT mouse model (ALK1HEC-KO) with liver sinusoidal endothelial cell (LSEC)-specific *ACVRL1* deficiency revealed hepatic vascular malformations, disrupted liver metabolic zonation, and right ventricular volume overload (Schmid et al., 2023). These changes were driven by the expression of proangiogenic and arterialization genes at the expense of LSEC and central venous identity. ALK1 signaling via BMP9/ALK1/ID (inhibitors of DNA binding 1-3) was shown to reduce angiokines (Wnt2, Wnt9b) and upregulate protein doppel and placental growth factor, two key factors in liver pathology (Schmid et al., 2023). Thus, hepatic endothelial ALK1 signaling offers insights for developing targeted HHT therapies.

These studies collectively demonstrate the multifaceted roles of BMPs in liver function, ranging from metabolic regulation to anti-fibrotic and anti-inflammatory effects. BMPs hold significant promise as therapeutic targets and biomarkers for conditions such as MASLD, MASH, and liver fibrosis (Tables 1–3), warranting further exploration to optimize their clinical applications.

### 2.5 GDFs

The GDFs are part of the BMP subfamily (Katagiri and Watabe, 2016). In the liver, the most prominent GDF is GDF15, which plays critical roles in liver physiology and pathology (Chung et al., 2017; Galuppo et al., 2022; Li et al., 2024). Recent findings highlight its diagnostic and therapeutic potential in liver diseases. Hepatocyte-specific overexpression of GDF15 in high-fat diet (HFD)-fed mice resulted in high circulating GDF15 levels, which improved obesity and hepatic steatosis (Takeuchi et al., 2024). The mechanism involved GDF15-enhanced splicing of X-box binding protein 1 (XBP1) and upregulation of the ER stress-related pathway, further promoting FGF21 expression by reducing CNOT6L levels (Takeuchi et al., 2024). On the other hand, high serum GDF15 levels were reported to be predictors of liver fibrosis in MASLD patients. Here, a novel non-invasive fibrosis index (MSI-F) incorporating GDF15 and decorin, effectively predicted fibrotic progression, underscoring GDF15’s utility as a biomarker in liver fibrosis assessment (Chang et al., 2024). Similarly, elevated GDF15 levels were significantly associated with poor survival in pediatric acute liver failure (PALF) (Zamora et al., 2024). GDF15 levels were higher in non-survivors than in survivors and were significantly lower in healthy children and patients with other liver diseases (Zamora et al., 2024). This suggests that GDF15 is a valuable prognostic biomarker for survival outcomes in PALF. Of note, GDF15 was also reported to have immunomodulatory effects on HSCs, suggesting its potential as a therapeutic target for liver fibrosis (Li et al., 2023). In conclusion, GDF15 has multifaceted roles in liver function and disease (Tables 1–3) and has the potential to serve both as a biomarker and as a therapeutic target for specific liver conditions.

### 2.6 GDNF and BAMBI

Glial cell line-derived neurotrophic factors (GDNFs) are considered a specific branch of the TGF-β-SF (Saarma, 2000; Karampetsou et al., 2022). GDNF is significantly upregulated in patients with advanced liver fibrosis and correlates with markers of HSC activation (Tao et al., 2019). In mice, GDNF overexpression exacerbated liver fibrosis, while silencing GDNF or blocking its signaling reduced fibrosis and HSC activation. It was shown that GDNF activates HSCs through binding to ALK5 at specific residues (His^39^ and Asp^76^) and inducing Smad2/3 signaling, independent of GDNF family receptor α-1 (Tao et al., 2019).

BMP and activin membrane-bound inhibitor (BAMBI) is a TGF-β pseudoreceptor that antagonizes TGF-β signaling (Onichtchouk et al., 1999), which is critical in inflammation and fibrogenesis (Weber et al., 2023). In liver fibrosis, BAMBI expression is downregulated in both rodent models and human patients, contributing to disease progression. Recent studies suggest that BAMBI overexpression can protect against liver fibrosis (Friedman, 2007; Weber et al., 2023). Moreover, BAMBI was found to have dual roles in HCC, demonstrating both tumor-promoting and tumor-protective effects (Weber et al., 2023). In addition, microRNA 942 (mir-942) expression was upregulated in activated HSCs and inversely correlated with BAMBI transcription and expression. TGF-β and lipopolysaccharide (LPS), key drivers of fibrosis and inflammation, induced mir-942 expression via Smad2/3 and NF-κB/p50 binding to its promoter (Tao et al., 2018). mir-942 degraded *BAMBI* mRNA, enhancing HSC sensitivity to TGF-β signaling and mediating LPS-induced proinflammatory responses (Tao et al., 2018).

Taken together, the studies described in Section 2 demonstrate the multiple roles of TGF-β-SF cytokines in liver function and numerous liver pathologies. The contribution of the different TGF-β-SF classes to liver diseases is summarized in Table 4.

**TABLE 4:** TGF-β superfamily cytokines in different liver pathologies.

S.N.	TGF-β superfamily cytokine	Liver pathology	References
1	TGF-β	Liver fibrosis and HCC	Fabregat and Caballero-Díaz (2018)
2	TGF-β	Liver fibrosis	Dewidar et al. (2019)
3	TGF-β/Activin A/BMP/GDF	Liver fibrosis, MAFLD/MASH and HCC	Wang, X. L. et al. (2024)
4	TGF-β	Liver fibrosis, MAFLD/MASH and HCC	Dooley and ten Dijke (2012)
5	TGF-β	Liver fibrosis	Yang et al. (2017)
6	TGF-β	Liver fibrosis, MAFLD/MASH and HCC	Giannelli et al. (2016b)
7	TGF-β	Liver fibrosis	Bertolio et al. (2021)
8	TGF-β	Liver fibrosis	Crouchet et al. (2025)
9	TGF-β	Liver fibrosis	Liu, X. et al. (2024)
10	TGF-β	Liver fibrosis, MAFLD/MASH	Ishiyama et al. (2024)
11	TGF-β	Liver fibrosis	Fan et al. (2019)
12	TGF-β	Liver fibrosis	Link et al. (2024)
13	TGF-β	Liver fibrosis, MAFLD/MASH, HCC	Dropmann et al. (2016)
14	TGF-β	Liver fibrosis	Jung et al. (2021)
15	TGF-β	Liver fibrosis, HCC	Color-Aparicio et al. (2024)
16	Activin A	Liver fibrosis, MAFLD/MASH, HCC	Rodgarkia-Dara et al. (2006)
17	Activin A	Liver fibrosis, MAFLD/MASH and HCC	Hamang et al. (2023)
18	Activin A	Liver fibrosis	Mekala et al. (2024)
19	BMP	Liver fibrosis	Bi and Ge (2014)
20	BMP	Liver fibrosis	Herrera et al. (2018)
21	BMP	Liver fibrosis	Tang et al. (2020)
22	BMP	Liver fibrosis, MAFLD/MASH and HCC	Chen et al. (2024)
23	BMP	Liver fibrosis	Breitkopf-Heinlein et al. (2017)
24	BMP	Liver fibrosis	Zou et al. (2019)
25	GDF	Liver fibrosis	Chung et al. (2017)
26	GDF	Liver fibrosis, MAFLD/MASH and HCC	Li et al. (2024)
27	GDF	Liver fibrosis	Li et al. (2023)
28	GDNF	Liver fibrosis	Tao et al. (2019)
29	BAMBI	Liver fibrosis, MAFLD/MASH and HCC	Weber et al. (2023)
30	BAMBI	Liver fibrosis	Tao et al. (2018)
31	TGF-β	Liver fibrosis, HCC	Yang et al. (2013)
32	TGF-β	Liver fibrosis	Cho et al. (2021)
33	TGF-β	Liver fibrosis	Tang et al. (2017)
34	TGF-β	Liver fibrosis	Palumbo-Zerr et al. (2015)
35	BMP	Liver fibrosis	Liu, B. et al. (2024)
36	TGF-β	Liver fibrosis	Mu et al. (2016)
37	TGF-β	Liver fibrosis	Wiercinska et al. (2006)
38	TGF-β	Liver fibrosis	Kasahara et al. (2023)
39	TGF-β	Liver fibrosis	Yu et al. (2022)
40	TGF-β	Liver fibrosis	Pan et al. (2024)
41	TGF-β	Liver fibrosis, MAFLD/MASH and HCC	Munker et al. (2017)
42	TGF-β	Liver fibrosis	Song et al. (2023)
43	TGF-β	Liver fibrosis	Yan et al. (2021)
44	TGF-β	Liver fibrosis	Rodríguez et al. (2021)
45	TGF-β	Liver fibrosis	Hui et al. (2021)
46	TGF-β	Liver fibrosis	Karkampouna et al. (2016)
47	TGF-β	MAFLD/MASH	Nair and Nath (2020)
48	TGF-β	MAFLD/MASH	Yang et al. (2014)
49	TGF-β	MAFLD/MASH	Yamaguchi et al. (2022)
50	TGF-β	MAFLD/MASH	Liu et al. (2022)
51	Activin A	MAFLD/MASH	Yndestad et al. (2009)
52	Activin A	MAFLD/MASH	Liu, H. et al. (2023b)
53	BMP	MAFLD/MASH	Yang et al. (2024)
54	BMP	MAFLD/MASH	Sun et al. (2021)
55	BMP	MAFLD/MASH	Li et al. (2019)
56	BMP	MAFLD/MASH	Wang et al. (2022)
57	GDF	MAFLD/MASH	Galuppo et al. (2022)
58	GDF	MAFLD/MASH	Takeuchi et al. (2024)
59	GDF	MAFLD/MASH	Chang et al. (2024)
60	TGF-β	MAFLD/MASH	Bai et al. (2024)
61	TGF-β	Liver fibrosis, MAFLD/MASH	Ahmed et al. (2022)
62	TGF-β	MAFLD/MASH	Chen, P. et al. (2018)
63	TGF-β	MAFLD/MASH	Liu et al. (2020)
64	TGF-β	Liver fibrosis	Masuda et al. (2020)
65	TGF-β	HCC	Katz et al. (2013)
66	TGF-β	HCC	Marvin et al. (2020)
67	TGF-β	HCC	Rao and Mishra (2019)
68	TGF-β	HCC	Gonzalez-Sanchez et al. (2021)
69	Activin A	HCC	Deli et al. (2008)
70	Activin A	HCC	Kreidl et al. (2009)
71	Activin A	HCC	Frost et al. (2011)
72	BMP	HCC	Herrera et al. (2014)
73	TGF-β	HCC	Hernanda et al. (2015)
74	TGF-β	HCC	Xin et al. (2024)
75	TGF-β	HCC	Feng, X. et al. (2015)
76	TGF-β	HCC	Majumdar et al. (2012)
77	TGF-β	Liver fibrosis, HCC	Yang et al. (2013)
78	TGF-β	HCC	Zhang et al. (2019)
79	TGF-β	HCC	Mohamed et al. (2024)
80	TGF-β	HCC	Zhang et al. (2006)
81	TGF-β	MAFLD/MASH	Gaitantzi et al. (2018)
82	TGF-β	HCC	Kiyono et al. (2009)
83	TGF-β	HCC	Senturk et al. (2010)
84	TGF-β	HCC	Ríos-López et al. (2023)
85	TGF-β	HCC	Meindl-Beinker et al. (2012)
86	TGF-β	HCC	Arrese et al. (2018)
87	TGF-β	HCC	Chen, J. et al. (2018)
88	TGF-β	HCC	Moon et al. (2017)
89	TGF-β	HCC	Xiao et al. (2025)
90	TGF-β	MAFLD/MASH, HCC	Dropmann et al. (2024)
91	TGF-β	HCC	Yin et al. (2016)
92	TGF-β	HCC	Koudelkova et al. (2017)
93	TGF-β	HCC	Moreno-Càceres et al. (2017)
94	TGF-β	HCC	Giannelli et al. (2020)
95	TGF-β	HCC	Deshmukh et al. (2021)
96	TGF-β	HCC	Sun et al. (2024)
97	TGF-β	HCC	Faivre et al. (2019)
98	TGF-β	HCC	Cuesta et al. (2023)
99	TGF-β	Liver fibrosis	Hammad et al. (2018)
100	TGF-β	HCC	Rani et al. (2015)
101	TGF-β	HCC	Kelley et al. (2019)
102	TGF-β	HCC	Harding et al. (2021)

## 3 TGF-β signaling pathways in liver disease: canonical and non-canonical

### 3.1 TGF-β signaling pathways

**FIGURE 1:**
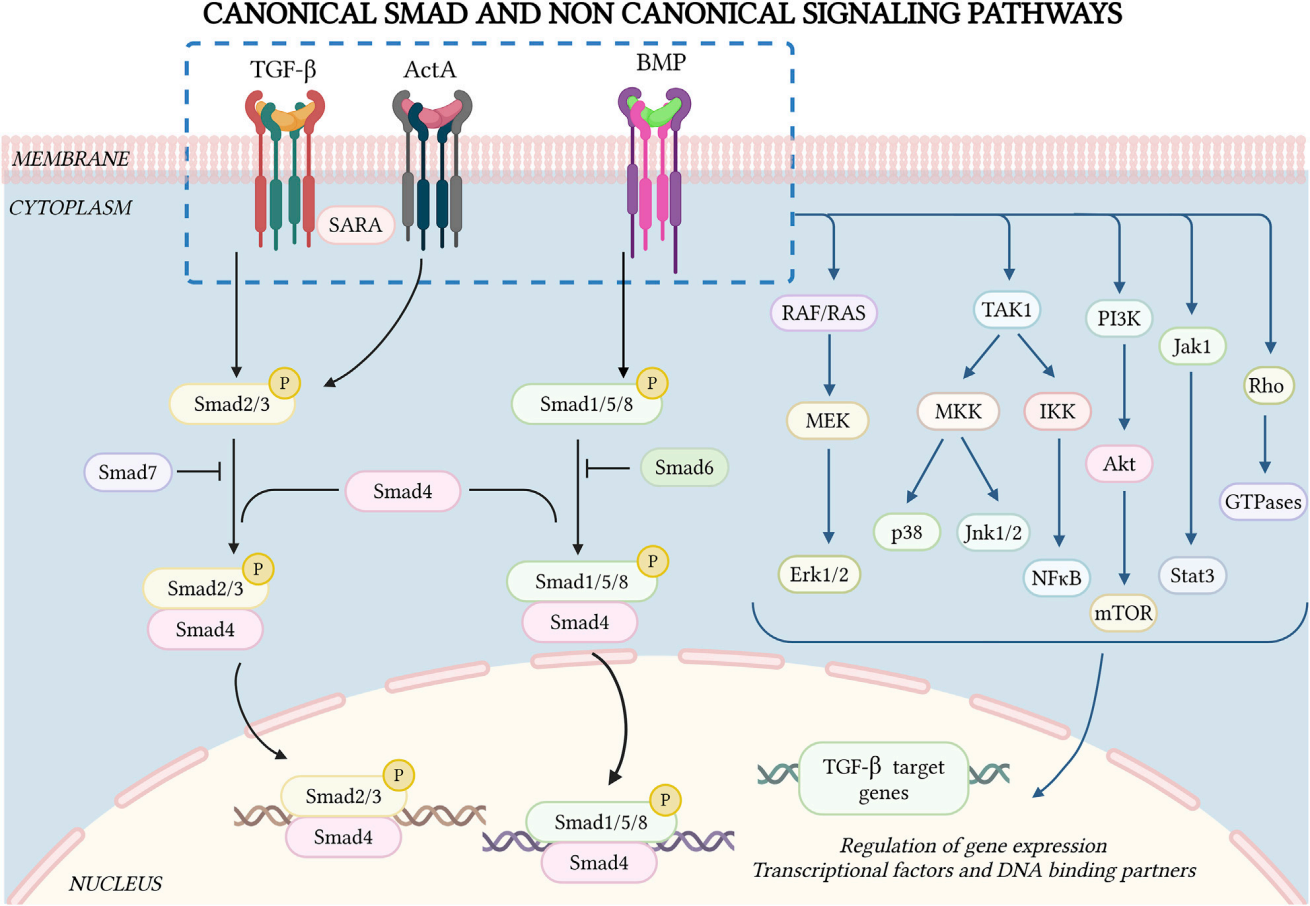
Canonical Smad and non-Smad signaling by TGF-β superfamily receptors. TGF-β-SF cytokine signaling is initiated by ligand binding to heteromeric type II and type I receptors, forming a signaling receptor complex. The type II receptor phosphorylates the relevant type I receptors in the complex, inducing C-terminal phosphorylation of specific Smad proteins (Smad2/3 for TGF-β and activins, Smad1/5/8 for BMPs). These then complex with Smad4, undergo nuclear translocation and together with other co-factors and DNA-binding partners activate or inhibit the transcription of multiple genes. Additionally, there are inhibitory Smads (Smad6/7) that downregulate Smad signaling through feedback mechanisms. TGF-β-SF cytokines also exhibit non-Smad signaling pathways, which vary depending on the cellular context. Some of these pathways (Jnk, p38, and NF-κB) are regulated by TAK1. Other central non-Smad pathways include Raf/Ras, Mek/Erk, PI3K/Akt and Rho GTPases. Figure drawn by BioRender.com on 26 January 2025.

TGF-β-SF cytokines exert pleiotropic effects in the liver, mediated via canonical and non-canonical pathways to promote diverse signaling outcomes (Figure 1). Their signaling is initiated via heterotetrameric complexes of type II/type I dual-specificity (Ser/Thr and Tyr) kinase receptors (Ehrlich et al., 2012; Goebel et al., 2019), activating canonical Smad (Smad2/3 or Smad1/5/8) and non-Smad pathways, whose crosstalk is crucial for multiple responses (Moustakas and Heldin, 2005; Yamashita et al., 2008; Parvani et al., 2011; Mu et al., 2012; Luo, 2017; Huang et al., 2022). Smad signaling is initiated by ligand binding to the receptors. Type II activates type I, which phosphorylates receptor-specific Smads (Smad2/3 for TGF-β and activins, or Smad1/5/8 for BMPs and GDFs) at their carboxy-terminal end. The phosphorylated Smad proteins complex with Smad4, translocate to the nucleus and activate or repress target gene transcription (Heldin and Moustakas, 2012; Massagué, 2012; Yadin et al., 2016) (Figure 1). Smads can also undergo phosphorylation in the linker region via multiple intermediate serine/threonine kinases, modulating signaling via the canonical Smad pathways and providing independent signaling cues (Kamato et al., 2020; Yamaguchi et al., 2022). Of note, similar to the dichotomous effects of TGF-β-SF ligands in HCC (Table 3), Smad4 displays a dichotomous behavior; while positively associated with tumor suppression at the initial stages of HCC, it becomes a tumor promoter at the late stages (Hernanda et al., 2015; Xin et al., 2024). Smad4 was reported to promote HCC by suppressing CD8^+^ T cell infiltration and glycolytic activity through the CXCL10/mammalian target of rapamycin (mTOR)/lactate dehydrogenase A (LDHA) pathway (Xin et al., 2024). Hepatocyte-specific Smad4 deletion in mice reduced liver tumors, fibrosis, and myeloid-derived suppressor cell infiltration while enhancing anti-tumor immunity (Xin et al., 2024). Moreover, TGF-β/Smad4 regulated hepcidin expression, iron transport, and homeostasis in the liver (Wang et al., 2005), whereas hepatic Smad7 overexpression caused severe iron overload in mice (Lai et al., 2018). Furthermore, Smad7 played a role in regulating hepatocyte proliferation in injured mouse livers and showed a positive association with human HCC (Feng T. et al., 2015).

TGF-β-SF receptors also stimulate non-Smad pathways in a cell-context-dependent manner (Figure 1). TGF-β and BMPs have been shown to activate p38/c-Jun N-terminal kinases (Jnk1/2), mitogen-activated protein kinases (MAPK) pathways, nuclear factor κ-light-chain enhancer of activated B cells (NF-κB), extracellular signal-regulated kinase (Erk1/2; Ras-Erk-MAPK pathway), phosphatidylinositol-3-kinase (PI3K/Akt), and Rho GTPases (Moustakas and Heldin, 2005; Galliher and Schiemann, 2007; Yamashita et al., 2008; Neil et al., 2009; Mu et al., 2012; Hamidi et al., 2017; Luo, 2017). Some activin receptors are shared by TGF-βs and BMPs, and activins also mediate non-Smad signaling (Moustakas and Heldin, 2005; Luo, 2017; Zhang, 2017). Several non-Smad pathways induce pro-metastatic effects, such as EMT, enhanced cell migration/invasion, etc. (Bhowmick et al., 2001; Rahimi and Leof, 2007; Heldin et al., 2012; Massagué, 2012; Luo, 2017; Zhang, 2017).

TGF-β-activated kinase 1 (TAK1) can be activated by multiple pathways, including TGF-β cytokines, tumor necrosis factor (TNF), interleukin-1 (IL-1) and toll-like receptors (Landström, 2010; Sakurai, 2012; Yang et al., 2013). It has been shown to activate several non-Smad pathways, including Jnk1/2, p38 and IκB kinase (IKK), which activates nuclear factor-kappa B (NF-κB). These pathways regulate apoptosis, proliferation, differentiation, and ECM production (Massagué, 2008; Landström, 2010; Dooley and ten Dijke, 2012; Majumdar et al., 2012; Sakurai, 2012; Yang et al., 2013) (Figure 1). Deletion of *TAK1* in hepatocytes elevated TGF-β signaling via the type II receptor (*TGFβR2*) and phospho-Smad2/3, contributing to liver fibrosis, inflammation, and the development of HCC (Yang et al., 2013). The central role of this pathway was demonstrated by the finding that mice lacking both *Tak1* and *Tgfbr2* (or *Smad4*) showed reduced liver injury, fibrosis, and HCC (Yang et al., 2013). Moreover, transcriptomics analysis identified the suppressor of IKKε (*SIKE*) as a conserved potent negative regulator of MAPK activation, a key molecular signature in the progression from MASLD to MASH (Bai et al., 2024). SIKE directly interacted with TAK1 and TAK1-binding protein 2 (TAB2) to disrupt their binding and subsequent activation of TAK1-MAPK signaling, connecting TAK1 to MASH pathology (Bai et al., 2024). Another study identified upregulation of Regulated in development and DNA damage response 1 (REDD1) in activated HSCs and in TGF-β-treated LX-2 cells (Cho et al., 2021). Here, TGF-β induction of REDD1 was Smad-independent, and involved the c-jun/AP-1 pathway. On the other hand, REDD1 overexpression inhibited TGF-β-induced Smad-dependent fibrogenic responses (Cho et al., 2021). These findings were confirmed *in vivo*, where infection with a REDD1 adenovirus reduced liver injury and fibrosis in a CCl_4_-induced mouse model (Cho et al., 2021). Notably, there are several lines of evidence implicating the TAK1-mediated NF-κB pathway in liver disease. For example, it has been reported that NF-κB induces the transcription of TGF-β1, which promotes ECM production in HSCs, leading to liver fibrosis (Feng X. et al., 2015). Furthermore, TGF-β upregulated nucleotide-binding oligomerization domain (Nod)-like receptor protein 3 (NLRP3) in HSCs, which activated the TAK1-NF-κB pathway, mediated the formation of inflammasomes and induced liver fibrosis (Kang et al., 2022). Interestingly, the tumor suppressive effects of TAK1 are mediated by NF-κB activation via the TNF pathway; this activation plays a critical role in preventing apoptosis of hepatocytes and cholangiocytes (Bettermann et al., 2010; Czauderna et al., 2019).

Another non-Smad pathway activated by TGF-β is the Janus kinase/signal transducer and activator of transcription (Jak-Stat) pathway, which is especially important for liver fibrosis. This pathway is indispensable for gene expression of a subset of TGF-β target genes in HSCs (Tang et al., 2017) (Figure 1). The pathway is stimulated via Jak1 through two distinct but complementary mechanisms. First, there is an early, rapid and direct Stat3 phosphorylation, which requires the Smad-independent binding of Jak1 to the type I TGF-β receptor (ALK5). Second, there is a late Stat3 activation that requires cooperativity with Smad2/3 activation (Tang et al., 2017). Together, these mechanisms amplify the pro-fibrogenic effects of TGF-β in the liver. In the context of hepatic cancer, TGF-β promotes tumor development through a newly identified H19 long noncoding RNA signaling axis via SRY (sex determining region Y)-box 2 [*SOX2*]. This axis specifically regulates tumor-initiating hepatocytes (TICs) while sparing the TGF-β responsiveness of other liver cells, both parenchymal and non-parenchymal. RNA sequencing (RNA-seq) analysis identified H19 as one of the most upregulated long noncoding RNAs (lncRNAs), in association with reducing the expression of the type II TGF-β receptor in TICs (Zhang et al., 2019). This signaling axis controls hepatic cancer development, and underscores the role of TGF-β signaling in shaping the tumor microenvironment and in driving cancer progression (Zhang et al., 2019).

An alternative pathway that can modulate non-Smad TGF-β signaling was reported to involve the orphan nuclear receptor NR4A1 (Palumbo-Zerr et al., 2015). This receptor was found to act as an endogenous inhibitor of TGF-β signaling, limiting TGF-β pro-fibrotic effects by recruiting a repressor complex to TGF-β target genes. Here, persistent TGF-β activation in fibrosis involved Akt and HDAC-dependent mechanisms, suppressing NR4A1 activation. Validation in animal studies was exemplified by the ability of small-molecule NR4A1 agonists to reduce fibrosis across multiple organs, including the liver (Palumbo-Zerr et al., 2015).

### 3.2 Receptor interactions and crosstalk between TGF-β-SF signaling pathways

Crystallographic studies on ligand-bound ectodomains of several type I/II TGF-β-SF receptors, including receptors for TGF-β, BMP and activin, suggested a heterotetrameric receptor structure (Allendorph et al., 2006; Weber et al., 2007; Groppe et al., 2008; Radaev et al., 2010; Townson et al., 2012; Goebel et al., 2019). Biophysical studies on the interactions between full-length TGF-β-SF receptors at the cell surface by immunofluorescence co-patching and patch/FRAP (fluorescence recovery after photobleaching) (Henis et al., 1994; Gilboa et al., 1998; Wells et al., 1999; Gilboa et al., 2000; Nohe et al., 2002; Rechtman et al., 2009; Marom et al., 2011; Ehrlich et al., 2012; Szilágyi et al., 2022) have shown that these receptors form heteromeric (type I/II) and homomeric (I/I or II/II) complexes without ligand (preformed complexes, PFCs), with ligand binding increasing mainly heterocomplex formation (ligand-mediated complexes, LMCs). These receptor complexes may be affected by the membrane composition, depending on cholesterol, which determines the formation of cholesterol/sphingolipid enriched domains (lipid rafts), of which caveolae are a subset (Hancock, 2006; Eisenberg et al., 2011; Parton and del Pozo, 2013). Among TGF-β-SF receptors, raft association was measured mainly for TGF-β and BMP receptors. They were found to partition between raft and non-raft domains, with higher raft fractions for type I receptors (Razani et al., 2001; Di Guglielmo et al., 2003; Hartung et al., 2006; Zuo and Chen, 2009; Guzman et al., 2012; Mundy et al., 2018). Reduced cholesterol level or knockdown of caveolin 1 (*CAV1*) were reported to inhibit TGF-β1 and BMP2-mediated non-Smad signaling (Erk1/2, Akt, p38), with no effect on the Smad pathways (Hartung et al., 2006; Meyer et al., 2011; Guzman et al., 2012; Shapira et al., 2014; Mundy et al., 2018). Recent studies in murine hepatocytes demonstrated remarkable differences between the effects of cholesterol on lipid raft localization of TGF-β receptors on Smad vs. non-Smad (Akt) signaling (Chaudhary et al., 2024). Preformed type I/II TGF-β receptor complexes were found to be cholesterol-dependent, and required lipid rafts to form.

Of note, Smad2/3 phosphorylation was independent of the cholesterol level and raft localization of the receptors, while non-Smad pAkt signaling was modulated by cholesterol in a time-dependent manner: it was enhanced by cholesterol depletion at short stimulation times, but reduced after prolonged stimulation. On the other hand, excess cholesterol (cholesterol enrichment) inhibited pAkt signaling by directly affecting this pathway. These findings imply that the cholesterol level modulates the balance between Smad and non-Smad (Akt) signaling by TGF-β in hepatocytes. This crosstalk between cholesterol and the balance between TGF-β signaling to Smad vs. non-Smad pathways has potential implications for hepatic diseases and malignancies.

The importance of the balance between Smad2/3 and non-Smad signaling in HCC is demonstrated by a recent study (Mohamed et al., 2024). In this study, inhibition of ALK5 by the kinase inhibitor LY2157299 disrupted Smad2/3 signaling (supposedly leading to tumor suppression) but not non-Smad signaling via the toll-like receptors (TLRs, contributing to tumor progression) (Mohamed et al., 2024). This interference with the balance between the Smad and non-Smad signaling arms of TGF-β abolished the cytostatic effects of TGF-β1 and led to the induction of IL-1 receptor-associated kinase (IRAK1). On the other hand, overexpression of ALK5 and knockdown of IRAK1 augmented the cytostatic effects of TGF-β1 in HUH-7 cells (Mohamed et al., 2024). Based on these results, it was proposed that disruption of this balance by inhibition of the canonical pathway induces HCC proliferation through TLR signaling.

TGF-β1 and BMP signaling also display functional crosstalk with each other in LSECs. These cells release BMP2, BMP6 and TGF-β1, which induce paracrine stimulation of hepatocytes and HSCs to control systemic iron homeostasis and fibrotic processes, respectively (Colucci et al., 2021). However, these cytokines also demonstrated an interactive autocrine signaling pattern in LSECs, where activation by TGF-β1 was retained, but not for BMP2 or BMP6 despite their high expression level. The loss of the response to BMP2/6 occurred despite the presence of the respective receptors, partly due to inhibition by FK-506–binding protein 12. In addition, TGF-β1 suppressed BMP2 expression via ALK5 (Colucci et al., 2021). These findings point out potential druggable targets for iron overload diseases, such as hereditary hemochromatosis, β-thalassemia and CLD. The crosstalk between TGF-β and BMP is further evidenced by the recent demonstration that BMP7 signaling regulates activation of HSCs by TGF-β via Smad1/5/8 (Liu B. et al., 2024). In this study, microRNA 3074 (miR-3074) was upregulated in HSCs stimulated by TGF-β1, promoting fibrosis. miR-3074 directly targeted and suppressed BMP7, which counteracts fibrosis (Liu B. et al., 2024). On the other hand, BMP7 overexpression reduced the fibrotic effects of miR-3074 in HSCs, and in liver fibrosis induced by CCl_4_ *in vivo* (Liu B. et al., 2024). TGF-β1/BMP crosstalk is further supported by the identification of stoichiometry-dependent crosstalk between TGF-β1and BMP6 in hepatocytes (Chen et al., 2016). TGF-β1 increased hepcidin expression in hepatocytes via ALK5 and type II TGFβ receptors through activation of Smad1/5/8, which is usually activated by BMPs (Chen et al., 2016). BMP6 also activated hepcidin mRNA expression via Smad1/5/8. However, elevated Smad2/3 signaling decreased the TGF-β1-mediated elevation in hepcidin mRNA, while the BMP6-hepcidin signal was elevated. These results suggest crosstalk between the two cytokines (Chen et al., 2016).

Activation of canonical TGF-β/activin and BMP signaling pathways showed coordinated responses in a liver injury murine model of acetaminophen-induced hepatotoxicity. Here, TGF-β and BMP pathways, detected by fluorescent TGF-β or BMP response element reporters, were found to promote autophagy and tissue repair. Conversely, Smad7 overexpression inhibited TGF-β signaling, initially exacerbating acute liver histopathology, but ultimately accelerating tissue recovery (Stavropoulos et al., 2022). This emphasizes the complex role of functional interconnectivity and coordinated activation between TGF-β-SF pathways in liver regeneration and disease (Stavropoulos et al., 2022).

## 4 Context-dependent role of TGF-β signaling in liver disease progression

TGF-β signaling exhibits distinct and cell-specific roles in liver disease, often displaying opposing actions depending on the cellular context and disease state. This multifaceted nature of TGF-β signaling underlines the complexity of its role in liver diseases. For instance, while TGF-β was shown to have a pro-fibrotic effect in the progression of liver diseases such as MASLD, MASH and HCC (Tables 1–4), other studies have indicated that in liver epithelial cells, TGF-β has a minimal impact on fibrogenesis and hepatocarcinogenesis. However, it plays a critical suppressive role in cholangiocarcinoma formation by inhibiting the proliferation of hepatocyte-derived cholangiocytes (Mu et al., 2016).

### 4.1 Proapoptotic and antiproliferative actions

TGF-β is associated with numerous pathways that regulate cell survival and death. Depending on the cellular context, its resultant effects can be fine-tuned, modulated and regulated. In most cases, TGF-β displays a cytostatic effect, which is mediated through its ability to suppress key transcriptional factors regulating growth control. These include c-myc and cell differentiation inhibitors such as inhibitor of differentiation factors (Id1/2/3) (Massagué, 2008; Zhang et al., 2017). For instance, Id1 has been identified as a critical regulator of TGF-β induced trans-differentiation of HSCs in a rat liver model. This study demonstrated that TGF-β treatment leads to enhanced Id1 protein expression in HSCs, mediated by the ALK1/Smad1 pathway rather than by stimulation of Smad2/3 by ALK5 (Wiercinska et al., 2006). Here, Id1 is identified as the TGF-β/ALK1/Smad1 target gene in HSCs and represents a critical mediator of trans-differentiation that might be involved in hepatic fibrogenesis (Wiercinska et al., 2006).

Aside from its role in inhibiting cell proliferation, TGF-β also acts as a potent inducer of apoptosis for hepatocytes, especially in cirrhotic liver (Dooley and ten Dijke, 2012; Zhang et al., 2017). In addition, TGF-β promotes the expression of death-associated protein kinase (DAP-kinase) by a different mechanism, inducing caspase activation and programmed cell death by a yet unknown mechanism, resulting in hepatocellular death (Jang et al., 2002). The expanding pool of identified proapoptotic genes that are transcriptionally regulated by TGF-β in hepatocytes includes *GADD45B*, *BMF*, and *BCL2L11* (Yoo et al., 2003; Ramjaun et al., 2007). In HCC cells, the gene for programmed cell death protein 4 (*PDCD4*), which is thought to play a crucial role in apoptosis, was found to be modulated by TGF-β signaling (Zhang et al., 2006). Another illustration of the TGF-β apoptotic effect in hepatocytes occurs through the adaptor protein Daxx, enhancing Fas-mediated apoptosis through the Jnk pathway (Yang et al., 1997). In hepatocytes, Daxx is found to directly interact with the type II TGF-β receptor, leading to the activation of Jnk and enhanced apoptosis, similar to Fas-induced apoptosis (Perlman et al., 2001). Of note, as expression of the above proteins can be modulated by other pathways, such crosstalk is likely to influence the sensitivity to TGF-β-induced apoptosis in these cell types. Furthermore, the absence of a consistent molecular program for TGF-β-initiated apoptosis may suggest that the cell death decision requires an integrated interpretation of multiple signaling inputs. Taken together, these mechanisms highlight the context-dependent ability of TGF-β to mediate cell survival or cell death of hepatocytes and other liver cells.

Moreover, TGF-β-induced pro-apoptotic effects in hepatocytes were found to be amplified by alcohol, an phenomenon recapitulated in human HCC liver tissue treated *ex vivo* (Gaitantzi et al., 2018). Alcohol boosted the TGF-β pro-apoptotic gene signature and the underlying mechanism of pathway crosstalk, significantly increasing cell death through the balance between Smad and non-Smad/Akt signaling. Blocking GSK-3β, a downstream mediator of Akt, rescued the strong apoptotic response mediated by alcohol and TGF-β (Gaitantzi et al., 2018). This interaction was independent of alcohol metabolism or oxidative stress. These findings highlight a direct crosstalk between ethanol and TGF-β, potentially contributing to the progression of chronic alcoholic liver disease (Gaitantzi et al., 2018).

### 4.2 Autophagy and senescence induction

TGF-β has been shown to rapidly stimulate autophagy in HCC cells, although the precise mechanistic details are still elusive (Kiyono et al., 2009). It also induces cellular senescence, a state of permanent cell cycle arrest, in various cell types, including several types of transformed HCC cells (Debacq-Chainiaux et al., 2005; Yoon et al., 2005; Senturk et al., 2010; Cipriano et al., 2011; Minagawa et al., 2011; Lin et al., 2012). Another TGF-β-SF member, ActA, also plays a very important role in age-related hepatocyte senescence by upregulating the expression of Cyclin-dependent kinase 4 inhibitor B (also known as p15^INK4b^) (Menthena et al., 2011). Interestingly, the activity of TGF-β is also highly niche-specific. For example, non-myelinating Schwann cells within the bone marrow provide the active form of TGF-β that maintains HSC dormancy, suggesting a novel role for TGF-β in regulating liver stem cell dynamics and making this glial cell type an important component of the HSC niche (Yamazaki et al., 2011).

These insights underscore the complex, site-specific, and context-dependent nature of TGF-β signaling in liver disease progression, offering valuable perspectives on potential therapeutic targets and mechanisms tailored to specific cellular contexts.

### 4.3 TGF-β in MASLD/MASH pathogenesis

While the role of TGF-β1 in liver fibrosis is well established (Table 1), its effects on MASH are complex and not fully understood (Table 2). The combination of inflammatory stress, lipid accumulation, and mediators such as pro-inflammatory interleukins and TGF-β1 drives MASLD progression to MASH. However, although targeting TGF-β1 signaling is central to anti-fibrotic therapies, its potential to reverse MASLD remains uncertain (Ahmed et al., 2022). TGF-β signaling orchestrates a wide range of cellular processes in these liver pathologies through both Smad and non-Smad pathways, which often interact with each other as well as with other signaling cues. For instance, YAP and TGF-β signaling pathways were shown to cooperate in promoting the development and progression of MASLD (Chen P. et al., 2018). Another study highlighted the interplay between TGF-β and Hippo pathways, focusing on the transcriptional cofactor TAZ (*WWTR1*). Here, TGF-β induced TAZ expression through the Smad pathway in HepG2 liver cancer cells, modulating liver cancer progression by contributing to liver size control, regeneration, and oncogenesis (Ríos-López et al., 2023). Similar effects were reported to occur due to crosstalk between TGF-β and the TNFα pathway (Liu et al., 2020). As reported in this study, hepatocyte-specific overexpression of TNFAIP3 interacting protein 3 (TNIP3) attenuated MASH progression in two murine dietary models (Liu et al., 2020). Unlike its traditional role as an inhibitor of TNFAIP3, TNIP3 functioned by direct interaction with TAK1, preventing its ubiquitination and activation in response to metabolic stress. As TAK1 is involved in inflammation and fibrosis, these findings suggest a potential approach to manage TGF-β-dependent liver damage and fibrosis (Liu et al., 2020).

In the context of MASLD/MASH pathogenesis, the effects of an antidiabetic drug (pioglitazone) in alleviating liver fibrosis through modulation of TGF-β signaling have been explored (Kasahara et al., 2023). Pioglitazone enhanced the production of linoleic acid (LA) metabolites by the gut microbiota, particularly 10-hydroxy-cis-12-octadecenoic acid (HYA), reducing steatosis, inflammation, and fibrosis in mice. HYA treatment suppressed TGF-β-induced Smad3 phosphorylation and fibrogenic gene expression in HSCs, suggesting that LA metabolites are important for the anti-fibrotic action of pioglitazone (Kasahara et al., 2023).

### 4.4 TGF-β paradox in hepatocellular carcinoma

TGF-β is a key regulator in CLD, influencing all stages from initial injury to inflammation, fibrosis, cirrhosis, and HCC (Table 1–3). It promotes hepatocyte destruction, HSC activation, and ECM deposition. In HCC, TGF-β has a dual role: while acting as a tumor suppressor at early stages of the disease, it switches to a tumor promoter at later stages of cancer progression, enhancing invasiveness and metastasis (Table 3). This conversion is driven by the activation of hepatocytes survival signaling (Meindl-Beinker et al., 2012; Arrese et al., 2018; Gonzalez-Sanchez et al., 2021). Consistent with the dual role of TGF-β in HCC, BAMBI has also been shown to have dual roles in HCC, demonstrating both tumor-promoting and tumor-protective effects (Weber et al., 2023). TGF-β signaling shows promise in short-term animal models, but its effects in human liver disease are more complex, requiring precise intervention in the right cell type at the appropriate disease stage to achieve therapeutic benefit (Meindl-Beinker et al., 2012) (Tables 1–3).

Somatic mutations in TGF-β signaling genes were found in 40% of HCCs, with *SPTBN1* being frequently altered. Transcriptome analysis revealed distinct HCC subgroups with either TGF-β pathway activation or inactivation, the latter being associated with poorer patient survival (Chen J. et al., 2018). Upregulation of genes in this pathway contributed to inflammation and fibrosis, whereas downregulation accompanied loss of TGF-β tumor suppressor activity. TGF-β signaling patterns were also positively correlated with activation of DNA damage response and sirtuin signaling pathways, and *SPTBN1* knockdown in HepG2, SNU398, and SNU475 cells increased their sensitivity to DNA crosslinking agents and reduced cell survival (Chen J. et al., 2018).

TGF-β inhibition via Smad7 overexpression or Smad2/3/4 knockdown reduced liver tumor formation in mouse models with either activated RAS (HRAS^G12V^) and p53 knockdown, or activated RAS and TAZ (Moon et al., 2017). In these models, TGF-β signaling promoted *SNAIL* gene transcription, whose knockdown suppressed tumor formation while its ectopic expression restored tumorigenesis, suggesting a critical role in tumor growth. In human HCC, Snail expression correlated with TGF-β activation and promoted cell proliferation (Moon et al., 2017).

Recently, Major facilitator superfamily domain containing 2 (MFSD2A) has been shown to be downregulated in HCC and to be associated with poor prognosis. MFSD2A overexpression inhibited HCC cell proliferation, migration, invasion, and EMT *in vitro*, and reduced tumor growth *in vivo* by inhibiting TGF-β-mediated Smad signaling (Xiao et al., 2025).

WNT1-inducible signaling pathway protein 1 (WISP1), which is now named cellular communication signaling factor 4 (CCN4), has recently been identified as a TGF-β target gene in several cells of hepatocyte origin (including primary mouse hepatocytes, AML12 and HepaRG cell lines) (Dropmann et al., 2024). WISP1 is known to be involved in balancing pro- and anti-tumorigenic TGF-β effects at premalignant CLD stages. Tissue microarray analysis revealed a positive correlation between WISP1 expression and early stages of HCC (Dropmann et al., 2024). *WISP1* transcripts were primarily found in hepatocytes of cirrhotic, rather than tumorous, liver tissue. High *WISP1* expression was linked to improved survival outcomes, suggesting potential cooperation between TGF-β and WISP1 in human HCC (Dropmann et al., 2024).

Another interesting aspect of the roles of TGF-β in HCC is demonstrated by the differential effects of the liver-specific miR-122 on liver cancer metastasis in humans vs. mice (Yin et al., 2016). In mice, miR-122 targeted the type I TGF-β receptor, while in humans it targeted a noncanonical site in the TGF-β1 5′UTR. Switching of the target of miR-122 between the receptor and ligand altered the metastatic behavior of liver cancer cells in both species (Yin et al., 2016). Additionally, the study identified over 50 other miRNAs that regulate TGF-β ligand-receptor pairs, suggesting an evolutionarily conserved mechanism of gene regulation underlying species-specific physiological or pathological phenotypes (Yin et al., 2016).

The entry of malignant hepatocytes into blood vessels is a crucial step in the spreading and metastasis of HCC. Understanding the underlying mechanisms is essential for developing treatments to alleviate metastasis. The effects of TGF-β on vascular invasion were investigated in a model of hepatocellular transmigration, involving hepatic sinusoidal endothelial cells and malignant hepatocytes (Koudelkova et al., 2017). The combination of TGF-β stimulation with cues from cell-cell interactions facilitated the invasion of malignant hepatocytes into blood vessels (Koudelkova et al., 2017).

HCCs often overexpress CAV1, whose expression level may convert the response of HCCs to TGF-β from pro-apoptotic to tumor-promoting (Meyer et al., 2013). Thus, studies comparing HCC cell lines expressing different levels of CAV1 along with CAV1 knockdown or overexpression, demonstrated differential effects of TGF-β on these parameters depending on the CAV1 levels. These studies indicated that in HCC cell lines, CAV1 protects against TGF-β-induced apoptosis by EGF receptor transactivation and PI3K/Akt signaling while reducing NADPH oxidase and pro-apoptotic *BMF* transcription (Moreno-Càceres et al., 2017). Consistent, with this idea, *CAV1* expression positively correlated with *TGFB1* in HCC patient samples (Moreno-Càceres et al., 2017).

## 5 Crosstalk between TGF-β signaling and metabolic pathways in liver pathogenesis

TGF-β signaling intricately interacts with metabolic pathways, influencing cholesterol and glucose metabolism in the liver and shaping the progression of liver diseases. Therefore, it is not surprising that TGF-β signaling pathways and metabolic pathways in the liver are intertwined and affect liver pathogenesis.

### 5.1 Cholesterol metabolism

We have recently identified a novel reciprocal inhibitory regulation between TGF-β and cholesterol, where TGF-β suppressed the expression of key genes regulating cholesterol metabolism, reducing total cholesterol levels and lipid droplet accumulation (Wang S. et al., 2024). This metabolic shift facilitated EMT, cytoskeletal reorganization and hepatocyte apoptosis. On the other hand, excess cholesterol protected hepatocytes from these TGF-β1-induced effects, while cholesterol depletion mimicked the consequences of exposure to TGF-β1 (Wang S. et al., 2024). These findings implicate TGF-β1 as a metabolic regulator in hepatocytes, linking lipid metabolism alterations to cellular processes that drive fibrogenesis and hepatocyte death. They suggest a novel mechanism whereby TGF-β modulates cholesterol metabolism in hepatocytes, directly impacting hepatocyte survival, differentiation, and HSC activation. This notion is in line with a study where CAV1, which is linked to cholesterol metabolism, was found to influence the metabolic gene signatures in TGF-β-treated hepatocytes. TGF-β primarily suppressed genes involved in glutathione, cholesterol, fatty acid and amino acid metabolism, and CAV1 knockdown upregulated them (Han et al., 2020). Additionally, 3-Hydroxy-3-methylglutaryl-CoA reductase (HMGCR), a central enzyme in cholesterol biosynthesis targeted by statins, emerged as a leading candidate in a genome-wide CRISPR screening aimed at identifying TGF-β-induced drivers of liver fibrosis in HSCs (Yu et al., 2022). Another report revealed that disruption of cholesterol biosynthesis or statin treatment enhanced TGF-β1 expression, promoting EMT and a mesenchymal phenotype in pancreatic cancer (Gabitova-Cornell et al., 2020). In this study, patient samples revealed that activation of TGF-β signaling and EMT by cholesterol-lowering statins may promote the basal type of pancreatic ductal adenocarcinoma, conferring poor outcomes in patients (Gabitova-Cornell et al., 2020).

Taken together, these findings highlight the crosstalk between TGF-β, CAV1 and cholesterol metabolism, which take part in shaping TGF-β-mediated metabolic regulation in both normal and diseased liver (Han et al., 2020).

### 5.2 Glucose homeostasis

A recent report has explored a new reciprocal interplay between TGF-β signaling and glucose metabolism via Forkhead box protein O1 (FoxO1), revealing a pivotal role of TGF-β in liver energy homeostasis (Pan et al., 2023). This work revealed a positive correlation between hepatic TGF-β1 expression with obesity and insulin resistance in both mouse and human models. Moreover, they demonstrated that TGF-β1 deficiency in the liver ameliorates glucose and energy imbalances in diet-induced obesity, improving metabolic health. Conversely, overexpression of TGF-β1 exacerbated glucose dysregulation and metabolic dysfunction in obese mice (Pan et al., 2023). This reciprocal regulation between TGF-β1 and FoxO1 highlights a critical axis controlling glucose metabolism and suggests a mechanism through which TGF-β signaling contributes to metabolic pathologies in the liver. For instance, FoxO1 promoted TGF-β1 expression and liver fibrosis in a CCl_4_-induced mouse model (Pan et al., 2024). Hepatic FoxO1 upregulation enhanced TGF-β1-mediated HSC activation and inflammation, contributing to fibrosis progression. These findings position FoxO1 as a critical upstream regulator of TGF-β1 in liver fibrosis (Pan et al., 2024).

The findings on TGF-β/cholesterol and TGF-β/glucose homeostasis highlight the profound impact of TGF-β signaling on hepatic metabolic processes, emphasizing its role as a key regulator of cholesterol and glucose metabolism. This crosstalk not only influences liver health but also contributes to broader metabolic dysfunctions, presenting potential therapeutic opportunities based on targeting TGF-β-mediated pathways in metabolic and liver diseases.

### 5.3 Insulin resistance

Several reports have indicated that TGF-β signaling is crucial for the development of insulin resistance, particularly under conditions of metabolic stress such as a high-fat diet (HFD). Using *Drosophila* as a model organism, it was shown that the expression of the glass bottom boat gene (*GBB*; a homolog of mammalian TGF-β in *Drosophila*) is elevated under HFD conditions, while its overexpression produces obese and insulin-resistant phenotypes (Hong et al., 2016). Inhibition of the Gbb pathway ameliorated HFD-induced metabolic phenotypes by promoting insulin resistance, mediated by increasing the expression of the tribbles gene (*TRB3*), a key inhibitor of insulin signaling (Hong et al., 2016). Elevated Trb3 protein levels impaired insulin-induced Akt phosphorylation in hepatocytes, disrupting glucose uptake and energy homeostasis. Importantly, dominant negative type I or type II TGF-β receptors could partially reverse these metabolic abnormalities, highlighting TGF-β signaling as a potential target for mitigating insulin resistance (Hong et al., 2016). Together, these results indicate that HFD-induced TGF-β/Gbb signaling provokes insulin resistance by increasing tribbles expression (Hong et al., 2016).

A further connection between insulin resistance and TGF-β signaling in the liver is implicated by the effects of insulin and TGF-β on the function of diseased liver. In end-stage liver disease, the loss of hepatocyte nuclear factor 4α (HNF4α) under inflammatory conditions correlates with impaired liver function and increased mortality. The level of HNF4α under these conditions is regulated by insulin sensitivity, and indirectly by TGF-β signaling through Smad2/3 and interaction with CCAAT/enhancer-binding protein α (C/EBPα). These results are reinforced by data from patients, where individuals expressing hepatic HNF4α also exhibited phospho-Smad2 and C/EBPα, while those lacking HNF4α showed deficiencies in either phospho-Smad2 or C/EBPα (Feng et al., 2024).

### 5.4 EMT and trans-differentiation in hepatocytes

Beyond its impact on insulin signaling, TGF-β contributes to broader liver pathophysiology through its ability to induce EMT in hepatocytes and to activate HSCs into myofibroblasts. Both of these processes drive liver fibrosis, linking TGF-β to progressive liver damage. Moreover, TGF-β signaling plays a dynamic role in various stages of liver disease, from initial injury to fibrosis, cirrhosis, and ultimately hepatocarcinogenesis (Giannelli et al., 2016a; Munker et al., 2017) (Tables 1–3). TGF-β can regulate the directional migration of hepatocyte cohorts, emphasizing its impact on adhesive behavior and motility. In response to TGF-β, hepatocytes undergo changes in their adhesion properties, particularly in integrin and fibronectin expression, which alter their migration patterns and modulate their motility and tissue remodeling (Binamé et al., 2008).

EMT-like changes observed in chronic liver disease may result from high TGF-β levels and other pro-fibrotic mediators, rather than by a direct conversion of parenchymal cells into myofibroblasts. Thus, although TGF-β was shown to influence cellular plasticity and contribute to fibrotic signaling pathways, these effects do not necessitate complete EMT *in vivo* at a time when essential liver functions are deteriorating (Munker et al., 2017). Another single-cell RNA sequencing study identified context-specific complexities of the EMT crosstalk and indicated that the Notch signaling pathway acted as a key driver of TGF-β-induced EMT. Furthermore, this study demonstrated that the gene signatures of pseudo-time clusters corresponding to the intermediate hybrid EMT state were associated with poor patient outcomes (Deshmukh et al., 2021). Additionally, ECM was found to have a pivotal role in shaping hepatocyte EMT responses to TGF-β. Thus, the response of hepatocytes to TGF-β depended on the stiffness of the matrix. Although TGF-β triggered the same transcriptional program, the outcome depended on growth matrix rigidity: EMT for cells growing on stiff collagen, and apoptosis for cells grown on soft collagen (Meyer et al., 2015). Therefore, the matrix environment appears to drive these divergent phenotypes rather than TGF-β signaling alone, offering important insights into fibrotic liver conditions (Meyer et al., 2015).

Studies on the dual role of TGF-β in regulating liver progenitor cells demonstrated that TGF-β-induced Smad signaling leads to growth inhibition and partial EMT, while TGF-β-MAPK signaling exerts opposing effects (Sun et al., 2024). Of note, phosphorylation of Smad3 at the linker region was critical in modulating these responses. The mutual antagonism between Smad3 and MAPK signaling in liver progenitor cells appears to enable these cells to overcome TGF-β-induced cytostasis under fibrotic conditions, supporting the maintenance of partial EMT and progenitor phenotypes (Sun et al., 2024).

Alagille syndrome is a condition associated with defective biliary development. In a mouse model of this disease, TGF-β signaling was demonstrated to have a role in driving hepatocyte transdifferentiation into cholangiocytes, an important process in the regeneration of functional bile ducts (Schaub et al., 2018). Unlike in traditional bile duct development, this process occurred in the disease model independently of canonical Notch signaling and represented a stable form of hepatocyte plasticity that persisted after cholestatic injury was resolved. These findings underscore the important roles of TGF-β in liver remodeling and its therapeutic potential in treating cholestatic liver diseases by enhancing biliary regeneration or hepatocyte transplantation (Schaub et al., 2018).

### 5.5 Activation of HSCs

TGF-β is an important mediator of HSC activation. A recent study reported the development of a high-throughput, genome-wide CRISPR/Cas9 screening platform to identify HSC-derived mediators of TGF-β-induced fibrogenesis (Yu et al., 2022). This screening platform revealed potential drug targets for liver fibrosis mediated by TGF-β. Another study on HSC activation suggested a role for the tyrosine kinase receptor (TrkB) in TGF-β-induced liver fibrosis (Song et al., 2023). TrkB inhibited TGF-β/Smad signaling, reducing HSC activation and fibrogenesis in liver spheroids and mouse models (Song et al., 2023). TGF-β promoted TrkB degradation via Nedd4-2-mediated ubiquitination, linking TrkB to the fibrotic process. In addition, adenoviral vector-mediated overexpression of TrkB in HSCs or hepatocytes alleviated liver fibrosis in multiple models mitigating liver fibrosis (Song et al., 2023). In line with these reports, Gli2, a key transcription effector of the Hedgehog signaling pathway, promoted HSC activation through the upregulation of cyclin and TGF-β signaling. Gli2 was upregulated in CCl_4_-induced liver fibrosis and its conditional deletion in HSCs ameliorated CCl_4_-induced liver fibrosis and HSC activation (Yan et al., 2021).

In conclusion, by influencing insulin signaling, fibrogenesis, HSC activation, EMT and cellular plasticity, TGF-β signaling emerges as a pivotal mediator in liver pathogenesis, providing insights into the intricate interplay between metabolic dysregulation and the progression of liver disease.

### 5.6 Oxidative stress

TGF-β signaling across multiple types of liver cells, including hepatocytes, stellate cells, and macrophages plays significant roles in both liver regeneration and fibrosis by targeting the production of NADPH oxidases (NOXs) (Herranz-Itúrbide et al., 2021). The TGF-β/NOXs axis generates reactive oxygen species (ROS) that are crucial in signal transduction pathways (Vermot et al., 2021). Recent evidence suggests that NOXs mediate many of the effects of TGF-β. Conversely, NOXs can also regulate TGF-β activity (Eva et al., 2015).

TGF-β signaling plays major functions in the transition of MASLD to MASH and can worsen MASLD with a backdrop of liver fibrosis (Ahmed et al., 2022). In hepatocyte-specific TGF-β receptor type II-deficient mice, inhibition of TGF-β signaling reduced liver steatosis, inflammation, and fibrosis in a MASH model, whereas its activation in steatotic hepatocytes promoted lipid accumulation and cell death through Smad2/3 signaling and ROS production (Yang et al., 2014). In addition, sinomenine attenuated acetaminophen (APAP)-induced acute liver injury by decreasing oxidative stress and inflammatory response via TGF-β/Smad signaling in both *in vitro* and *in vivo* models (Chen et al., 2020). In the context of the effects of TGF-β on HSC activation and fibrogenesis through ROS production, a maleic acid derivative that effectively reduce ROS production, inflammatory response, and fibrotic markers by modulating TGF-β signaling has been identified (Yang et al., 2017). Interestingly, Maresin-1 (MaR1), a derivative of ω-3 docosahexaenoic acid (DHA), ameliorated liver fibrosis in a diethylnitrosamine-induced rat model by reducing oxidative stress induced by TGF-β. MaR1 inhibited the translocation of p65, a component of NF-κB, while promoting the activation of Nrf2, a key regulator of the antioxidant response that reduces oxidative stress and inflammation in this liver fibrosis model (Rodríguez et al., 2021).

### 5.7 Lipotoxicity

TGF-β signaling plays a critical role in the progression of lipotoxicity-associated liver diseases such as MASLD/MASH (Table 2). Lipotoxicity, characterized by the toxic accumulation of lipids in liver cells, triggers a cascade of genetic and epigenetic alterations mediated by TGF-β signaling in lipotoxicity-induced liver carcinogenesis (Table 2). Specifically, Smad3 isoforms phosphorylated at the linker region exhibit roles distinct from those of Smads phosphorylated at the C-terminus. Linker-phosphorylated pSmad3L promotes hepatocyte proliferation, contributing to liver carcinogenesis (Kamato et al., 2020; Yamaguchi et al., 2022). This exacerbates disease progression by suppressing the cytostatic effects of this signaling pathway. During chronic inflammation-mediated MASH pathogenesis, TGF-β signaling also drives the trans-differentiation of HSCs into myofibroblasts, a key event in liver fibrosis. This process links lipotoxicity-induced stress to structural and functional liver damage, underscoring the role of TGF-β as a central mediator at various stages of MASH pathogenesis. These findings highlight the intricate relationship between TGF-β signaling and lipotoxicity in liver disease progression, and point to potential therapeutic avenues by targeting specific Smad3 isoforms and TGF-β pathways to mitigate the impact of lipotoxicity in MASH and related conditions.

## 6 Therapeutic potential of targeting TGF-β signaling in liver diseases

Targeting TGF-β signaling offers promising potential for managing liver diseases such as fibrosis, cirrhosis and HCC (Tables 1–4). Recent advances highlight novel compounds and peptides with significant efficacy in mitigating pathological effects related to TGF-β signaling (Faivre et al., 2019; Giannelli et al., 2020; Cuesta et al., 2023). TGF-β signaling can be inhibited by several strategies, including monoclonal antibodies, vaccines, antisense oligonucleotides, and small molecule inhibitors. For instance, Oxy210, a dual inhibitor of Hedgehog and TGF-β, has been demonstrated to possess anti-fibrotic and lipid-regulating effects. Using a humanized hyperlipidemic MASH mouse model, oral Oxy210 administration significantly reduced hepatic fibrosis, plasma pro-inflammatory cytokines, pro-fibrotic gene expression, and apoptosis in the liver (Hui et al., 2021). This treatment also reduced hepatic cholesterol accumulation and plasma cholesterol levels. Another report suggested that inhibition of TGF-β signaling in hepatocytes enhances liver regeneration, as evidenced by increased expression of cell proliferation markers and improved recovery of the CCl_4_-metabolizing enzyme CYP2E1 (Karkampouna et al., 2016). It was concluded that aside from its impact on fibrosis and matrix remodeling, blocking TGF-β signaling also promotes liver cell regeneration, offering insights into potential therapeutic strategies for liver damage (Karkampouna et al., 2016). Similarly, galunisertib (LY2157299 monohydrate), a small-molecule inhibitor targeting the type I TGF-β receptor kinase, demonstrated both anti-fibrogenic and regenerative properties in a mouse model of CCl_4_-induced liver cirrhosis, suggesting its potential in treating advanced liver fibrosis (Masuda et al., 2020). In another preclinical model (Abcb4ko mice), galunisertib effectively reduced Smad2 phosphorylation in liver cells, indicating successful TGF-β pathway inhibition (Hammad et al., 2018). This drug downregulated the expression of fibrogenic genes and decreased ECM proteins such as fibronectin and laminin-332, as well as the β-catenin pathway (Hammad et al., 2018), which may promote carcinogenesis in some circumstances. Additionally, inhibition of TGF-β signaling was found to modulate stemness-related biomarkers in HCC cell lines and in human HCC tissue samples (Rani et al., 2015).

It should be noted that long-term usage of galunisertib may also pose risks. It was shown to cause cardiac toxicity in animals, a finding that initiated the development of a pharmacokinetic/pharmacodynamic-based dosing regimen to enhance its safety (Herbertz et al., 2015). Galunisertib is under investigation as monotherapy or in combination with different therapies for cancers with unmet treatment needs, including HCC. For instance, the therapeutic efficacy of the galunisertib/sorafenib combination in advanced HCC patients with preserved liver function was evaluated (Kelley et al., 2019). The combined treatment demonstrated an acceptable safety profile and improved outcomes, with an increased median overall survival (OS) (Kelley et al., 2019). Patients with over a 20% reduction in TGF-β1 levels had significantly longer OS, suggesting that galunisertib may enhance the therapeutic efficacy of sorafenib in HCC (Kelley et al., 2019). Another combination therapy of galunisertib and ramucirumab (anti-VEGF receptor 2 antibody) was undertaken in advanced HCC patients (Harding et al., 2021). Since preclinical data suggested that TGF-β and VEGF signaling interactions could synergistically promote angiogenesis and immune evasion, their combined inhibition is a potential therapeutic strategy for HCC (Harding et al., 2021). This phase Ib trial investigated the safety, tolerability, and maximum tolerated dose of galunisertib and ramucirumab in advanced HCC patients. Although this combination was safe and showed favorable pharmacokinetics, the objective response rate and the disease control rate were low and did not support the hypothesis that TGF-β inhibition enhanced VEGF-targeted therapy (Harding et al., 2021).

Given the limited success of targeting the TGF-β pathway with small molecule inhibitors like galunisertib, alternative strategies to modulate this pathway need to be developed. One promising approach involves the use of Z-RIPΔ, a novel TGF-β type I receptor-mimicking peptide that selectively binds to TGF-β1-activated HSCs, inhibiting proliferation, migration, and fibrosis marker expression (Liu X. et al., 2024). Z-RIPΔ specifically targets fibrotic liver tissue, improving liver histopathology *in vivo* with stronger anti-fibrotic effects than its parent peptide (Liu X. et al., 2024). Collectively, these findings underscore the potential of targeting TGF-β pathway modulators as effective therapeutic strategies for liver diseases, paving the way for targeted and precise interventions (Tables 1–4).

## 7 Concluding remarks

The liver is a prime example of the inherent complexity and context dependence of TGF-β-SF signaling, arising from an hourglass-like structure of the signaling pathways. This structure is characterized by a broad number of cytokines, a more restricted number of distinct receptor complexes that activate two sets of canonical Smad-dependent signaling pathways. On the other hand, the signaling outcomes also depend on signals from a broader number of non-canonical pathways and input from crosstalk with pathways activated by other hormones, which impact the expression of hundreds of genes. Ligand expression, the repertoire of non-canonical pathways, the crosstalk between pathways and the accessibility of target genes all heavily depend on cellular context. As a result, TGF-β-SF cytokines perform distinct and sometimes contradictory functions in liver homeostasis and disease progression. Throughout this review, we focused on how the balance between the multiple pathways affect critical functions in specific hepatocellular processes. Of particular interest are the contributions of TGF-β-SF to liver diseases, including MASLD, MASH, and HCC. In recent years, innovative therapeutic approaches, including inhibition of signaling by the application of soluble receptors, or activation via receptor mimetics, have been developed. These developments rely heavily on the understanding of the intricate dynamics of multiple TGF-β-SF signaling pathways and factors that modulate them in the liver. Manipulation or fine-tuning of these pathways should offer promising opportunities for the development of future therapies.
